# Supplementary material for: PDX1+ cell budding morphogenesis in a stem cell-derived islet spheroid system
Source: Nat Commun. 2024 Jul 13;15:5894. doi: 10.1038/s41467-024-50109-2 (PMC11246529; doi:10.1038/s41467-024-50109-2)
Supplement: Supplementary file 1 — Supplementary Information [file 41467_2024_50109_MOESM1_ESM.pdf]

Manuscript title:
PDX1+ cell budding morphogenesis in a stem cell-derived islet spheroid system

Authors:

Jia Zhao, Shenghui Liang, Haoning Howard Cen, Yanjun Li, Robert K. Baker, Balwinder Ruprai, Guang Gao, Chloe Zhang, Huixia Ren, Chao Tang, Liangyi Chen, Yanmei Liu, Francis C. Lynn, James D. Johnson and Timothy J. Kieffer

Contents:

Supplementary Fig. 1: Two types of differentiation patterns by fine tuning Wnt signaling at the endoderm stage.
Supplementary Fig. 2: Differentially expressed Wnt pathway genes in the endoderm cells generated by bulk- and budding-type differentiations.
Supplementary Fig. 3: Transcriptomic differences in the pancreatic, pro-endocrine and islet cells generated by bulk- and budding-type differentiations.
Supplementary Fig. 4: Reproducibility of budding spheroid differentiations with various Wnt agonists in multiple hPSC lines.
Supplementary Fig. 5: Quantification of bud formation efficiency in Wnt^{low}-mediated budding-type differentiations.
Supplementary Fig. 6: Representative images showing the whole clusters with both islet buds and main bodies.
Supplementary Fig. 7: Assessment of PDX1+ cell proliferation rate during budding process.
Supplementary Fig. 8: Spontaneous clustering of PDX1+ cells in both planar and suspension cultures.
Supplementary Fig. 9: The process of inducing differentiation is required for islet cell rearrangement.
Supplementary Fig. 10: Perifusion of single primary human islet cluster and detection of dynamic insulin secretion in a microfluidic chip system.
Supplementary Fig. 11: Additional morphological and functional phenotypes in *PDX1* KO and *RFX6* KO hESC cultures.
Supplementary Fig. 12: Purified islet buds continue to differentiate after separation from main bodies.
Supplementary Fig. 13: Additional characterization of main body cells generated by budding-type differentiation.
Supplementary Fig. 14: Screening of different signaling pathways on PDX1+ cell clustering.
Supplementary Fig. 15: Differential expression of EphB/EphrinB in endocrine buds and main bodies.
Supplementary Fig. 16: Inhibition of EphB3/4 signaling interrupts PDX1+ cell clustering.
Supplementary Fig. 17: Examination of EphB/EphrinB expression in Stage 5 clusters derived from *PDX1* KO hESCs.
Supplementary Fig. 18: Staining of adhesion molecules, EMT markers and cell polarity markers.
Supplementary Fig. 19: Validation of Mel1 $INS^{GFP/W}$ and HUES4 PDXeG reporter lines.
Supplementary Fig. 20: An example of gating strategy for flow cytometry experiments in this study.
Supplementary Table 1: Comparison of differentiation process and summary of Wnt^{low}-mediated budding-type and Wnt^{med}-mediated bulk-type systems.
Supplementary Table 2: Key reagents and resources used in this study.
Supplementary Table 3: The sequences of qPCR primers used in this study.

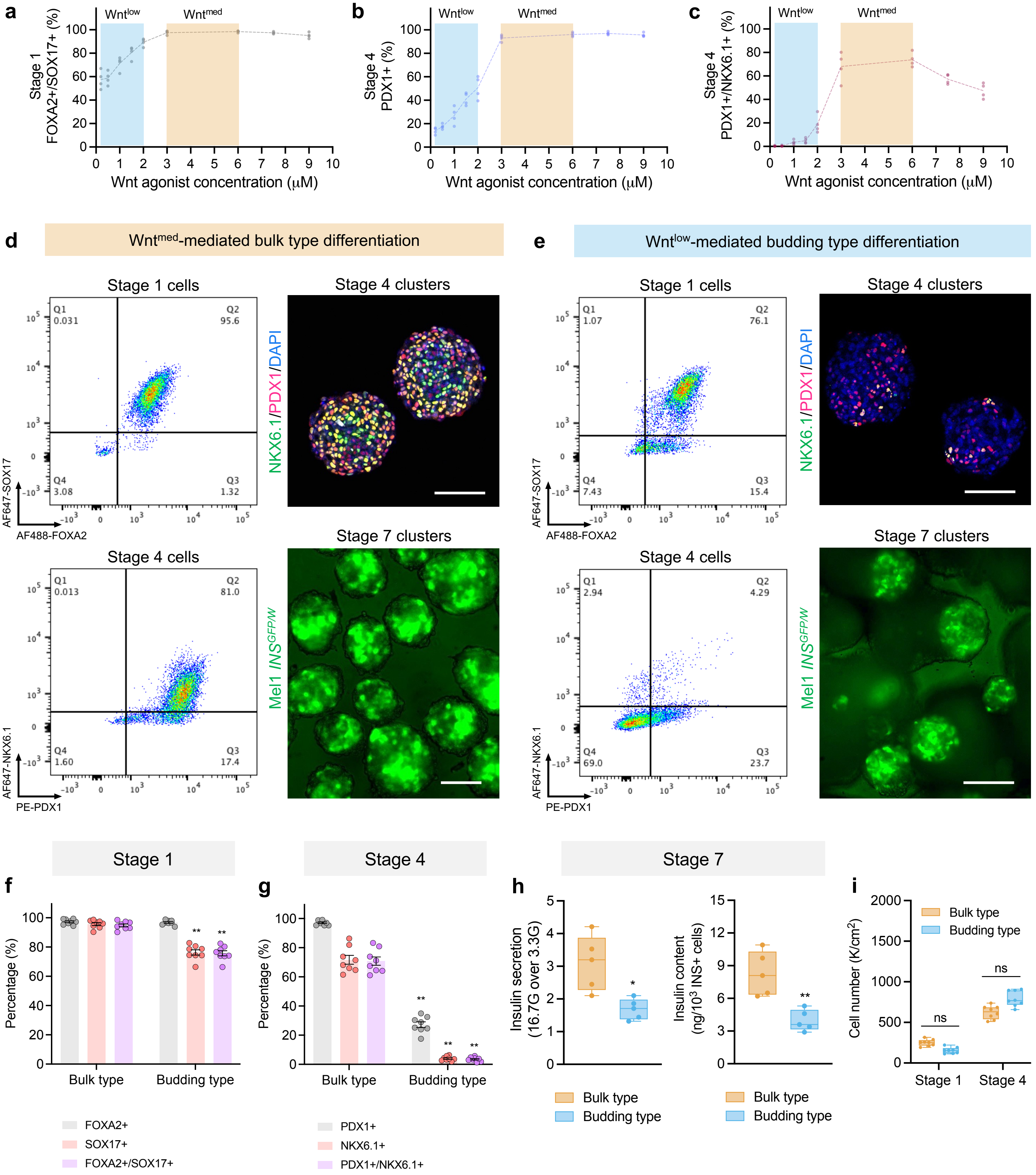

Supplementary Fig. 1: Two types of differentiation patterns by fine tuning Wnt signaling at the endoderm stage.
(**a-c**) The Wnt agonist CHIR99021 dose-ranging tests (in combination with 100 ng/mL GDF8) to define Wnt^{low} and Wnt^{med} conditions using HUES4 PDXeG line and Mel1 *INS*$^{GFP/W}$ line (n = 2 independent differentiations for each line). (**d-e**) Representative data at indicated stages from typical Wnt^{med}-mediated bulk-type differentiations (**d**) and Wnt^{low}-mediated budding-type differentiations (**e**). Scale bars, 100 μm. (**f-g**) Flow cytometry quantification showing Stage 1 (**f**) and Stage 4 (**g**) differentiation efficiencies in bulk and budding-type cultures, as indicated by DE markers (FOXA2, SOX17) and pancreatic progenitor markers (PDX1, NKX6.1), respectively. n = 8 independent differentiations, **p < 0.01 versus bulk type, unpaired two-tailed t-test. (**h**) Insulin secretion and total insulin content from bulk- and budding-type Stage 7 sorted INS+ cells. n = 5 independent differentiations, ns, not significant, **p < 0.01 versus bulk type, unpaired two-tailed t-test. (**i**) Quantification showing comparable cell numbers from bulk- and budding-type cultures at indicated stages. n = 8 independent differentiations, ns, not significant, unpaired two-tailed t-test. Bulk and budding differentiation were induced by 100 ng/mL GDF8 plus 3 μM and 1-1.5 μM CHIR99021 for Stage 1 Day 1, respectively, using the Mel1 *INS*$^{GFP/W}$ line in **1d-1i**.

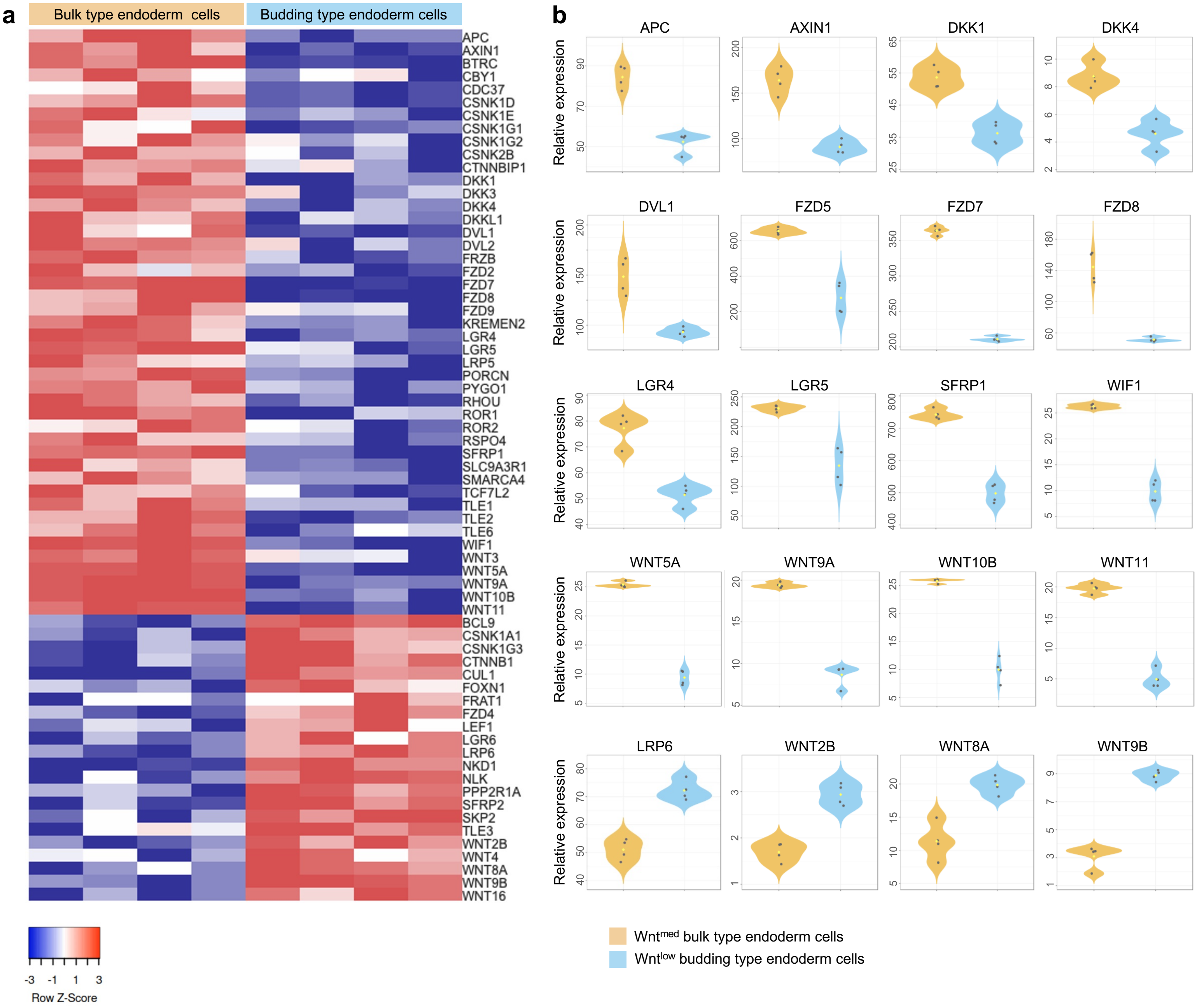

Supplementary Fig. 2: Differentially expressed Wnt pathway genes in the endoderm cells generated by bulk- and budding-type differentiations.
(**a**) The heatmap showing differentially expressed Wnt pathway genes in the definitive endoderm cells induced by Wnt^{low} and Wnt^{med} protocols. (**b**) Relative expression of the selected differential Wnt pathway genes in the definitive endoderm cells induced by Wnt^{low} and Wnt^{med} protocols. Data were normalized to housekeeping genes and the relative expression was presented by comparison to undifferentiated stem cell controls. Bulk and budding differentiation were induced by 100 ng/mL GDF8 plus 3 µM and 1-1.5 µM CHIR99021 for Stage 1 Day 1, respectively, using the Mel1 *INS*$^{GFP/W}$ line. n = 4 independent differentiations.

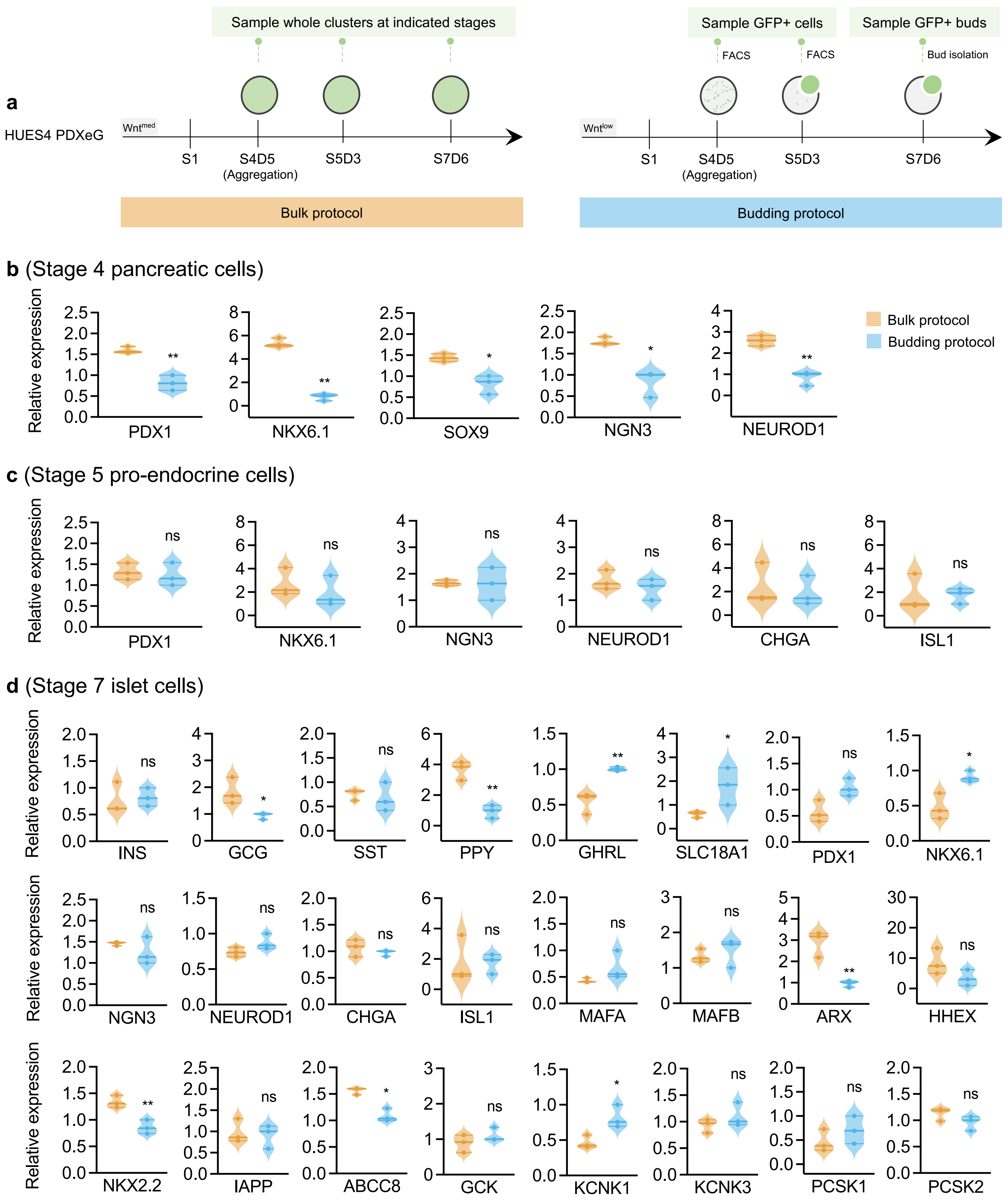

Supplementary Fig. 3: Transcriptomic differences in the pancreatic, pro-endocrine and islet cells generated by bulk- and budding-type differentiations.

(**a**) Experimental design for transcriptomic profiling of pancreatic cells, pro-endocrine cells and islet cells induced by bulk- and budding-type protocols. Created with BioRender.com released under a Creative Commons Attribution-NonCommercial-NoDerivs 4.0 International license. (**b-d**) Comparison of key transcripts in pancreatic cells (**b**), pro-endocrine cells (**c**) and islet cells (**d**) induced by the two protocols at indicated stages. Data were normalized to the housekeeping gene NFX1 and the relative expression was presented by comparison to the biological replicate #1 of budding-type cells. n = 3 independent differentiations, ns, not significant, *p < 0.05, **p < 0.01 versus bulk type, unpaired two-tailed t-test. Bulk and budding differentiation were induced by 100 ng/mL GDF8 plus 3 μM and 1.5 μM CHIR99021 for Stage 1 Day 1, respectively, using the HUES4 PDXeG line.

a

	CHIR99021 + 100 ng/mL GDF8		CHIR99021 + 100 ng/mL Activin A		MCX-928 + 100 ng/mL GDF8		mWnt3a + 100 ng/mL GDF8	
	Bulk	Budding	Bulk	Budding	Bulk	Budding	Bulk	Budding
Mel1 *INS*$^{GFP/W}$	CHIR99021 3-6 µM	CHIR99021 0.5-1.5 µM	CHIR99021 3 µM	CHIR99021 0.2-0.5 µM	MCX-928 1 µM	MCX-928 0.2-0.5 µM	NT	mWnt3a 25-50 ng/ml
H1 hESC	CHIR99021 3-6 µM	CHIR99021 0.2-0.5 µM	CHIR99021 3 µM	CHIR99021 0.2-0.5 µM	MCX-928 1 µM	MCX-928 0.1-0.2 µM	NT	mWnt3a 25-50 ng/ml
HUES4 PDXeG	CHIR99021 3-6 µM	CHIR99021 0.5-1.5 µM	NT	NT	MCX-928 1 µM	MCX-928 0.2-0.5 µM	NT	mWnt3a 25-50 ng/ml
GCaMP hiPSC	CHIR99021 3-4 µM	CHIR99021 0.5-1 µM	CHIR99021 3 µM	CHIR99021 0.5-1 µM	NT	NT	NT	NT

Note: As indicated above, the concentrations of Wnt agonists used to initiate budding type differentiation are cell line dependent. NT, not tested.

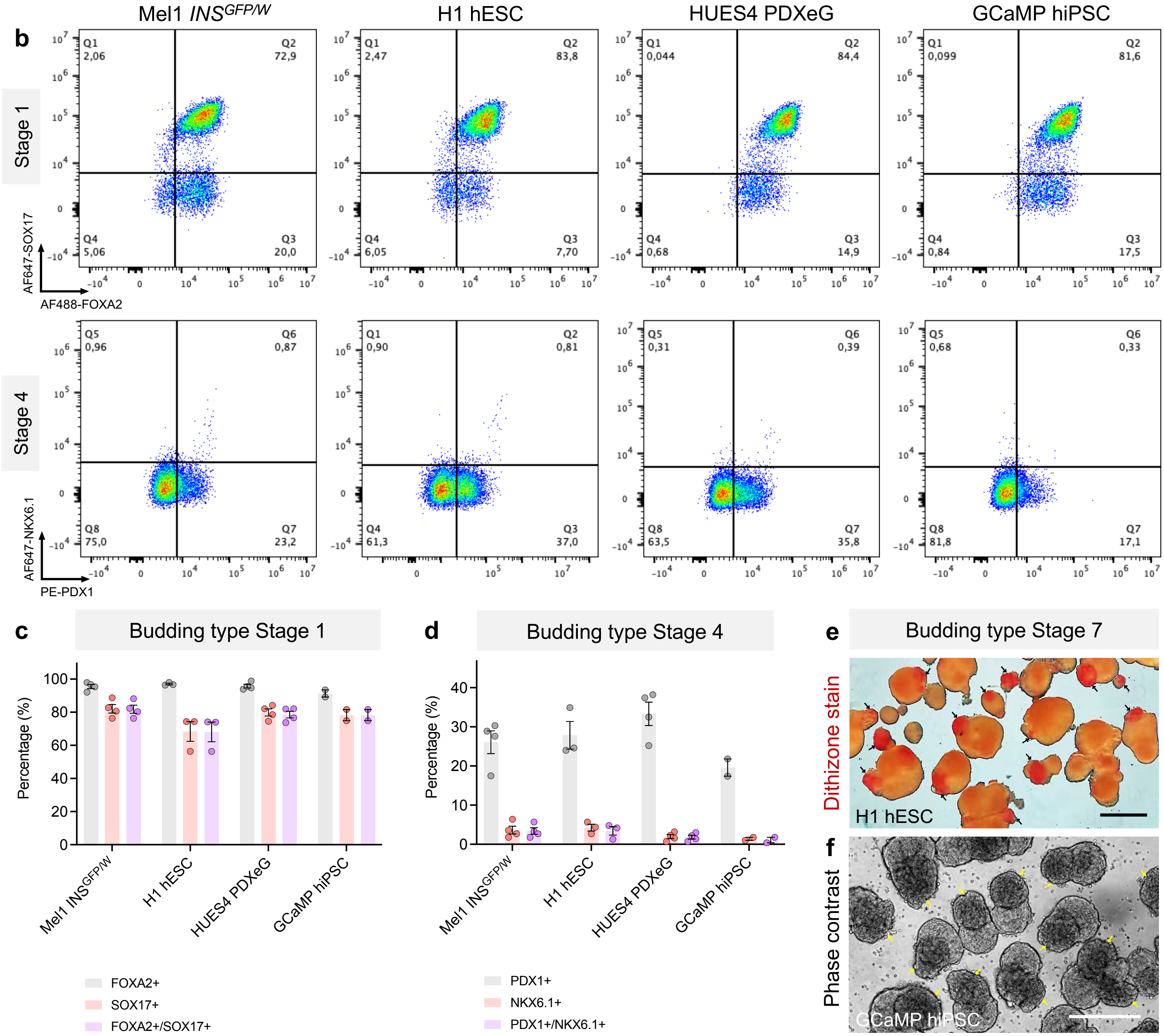

Supplementary Fig. 4: Reproducibility of budding spheroid differentiations with various Wnt agonists in multiple hPSC lines.

(**a**) Consistent induction of bulk- and budding-type differentiations in multiple hPSC lines with various Wnt agonists (CHIR99021, MCX-928, mWnt3a) in combination with GDF8 (or Activin A) only for the first day of the DE stage. For the next two days, cells were treated with GDF8 (or Activin A) without Wnt agonists. (**b**) Representative flow cytometry plots of Stage 1 cells (upper panels) and Stage 4 cells (bottom panels) from budding differentiation cultures derived from indicated hPSC lines. (**c-d**) Flow quantification showing Stage 1 (**c**) and Stage 4 (**d**) differentiation efficiencies in budding-type cultures, as indicated by DE markers (FOXA2, SOX17) and pancreatic progenitor markers (PDX1, NKX6.1), respectively. n = 2-4 independent differentiations. (**e**) Dithizone staining showing crimson red signals enriched in bud-like structures (indicated by arrows) in budding-type Stage 7 clusters derived from H1 cells. Scale bar, 200 µm. (**f**) Phase contrast image showing compact and dark colored bud-like structures (indicated by arrows) in budding-type Stage 7 clusters derived from GCaMP hiPSC cells. Scale bar, 200 µm. Budding differentiation was induced by 100 ng/mL GDF8 plus 1-1.5 µM CHIR99021 in Mel1 *INS*$^{GFP/W}$ line, 100 ng/mL GDF8 plus 0.2-0.5 µM CHIR99021 in H1 line, 100 ng/mL GDF8 plus 1-1.5 µM CHIR99021 in HUES4 PDXeG line, and 100 ng/mL GDF8 plus 0.5-1 µM CHIR99021 in GCaMP hiPSC line for Stage 1 Day 1.

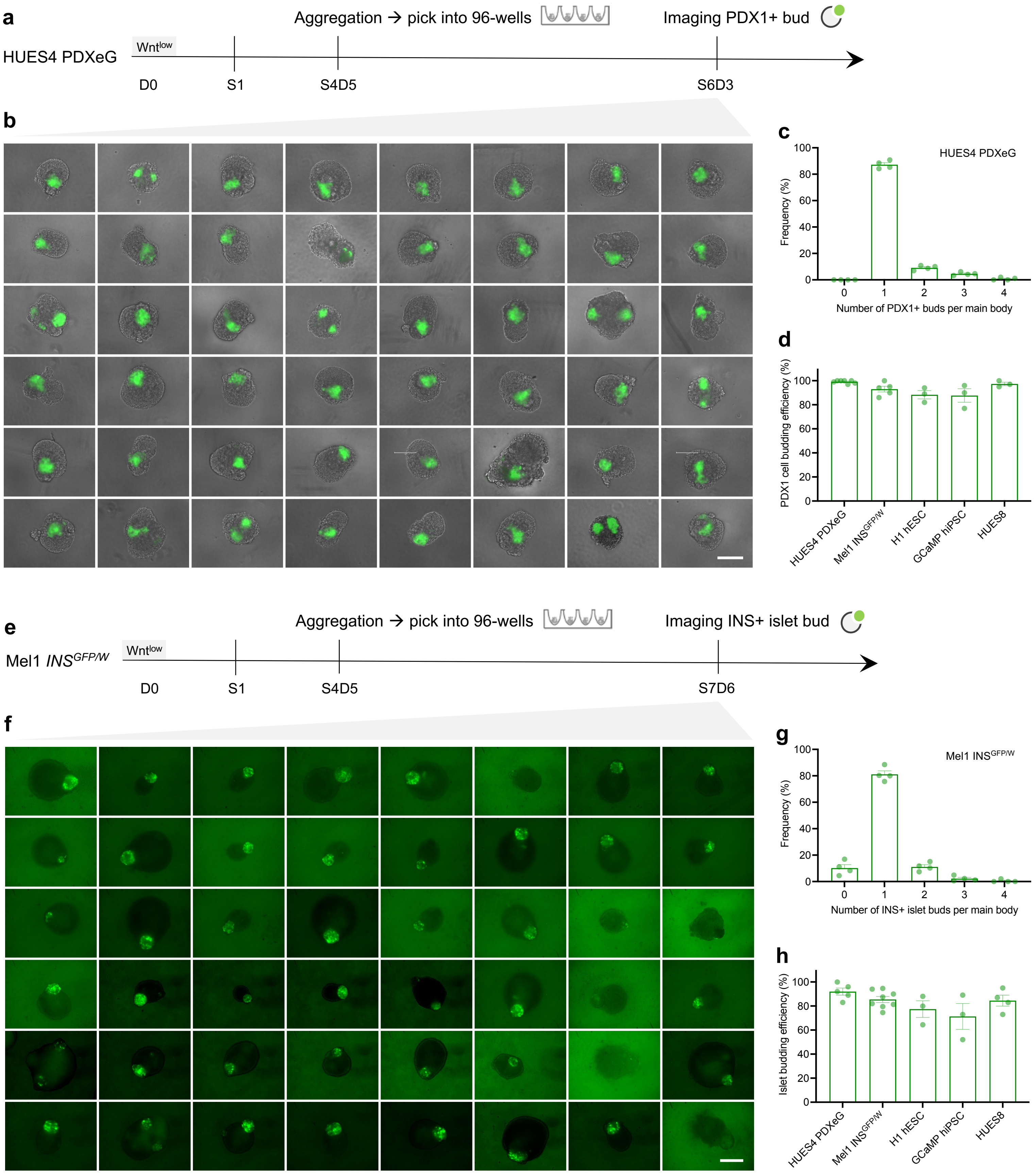

Supplementary Fig. 5: Quantification of bud formation efficiency in Wnt^{low}-mediated budding-type differentiations.
(**a-c**) Representative experiment showing PDX1+ bud formation in 96-well assays (**a-b**), and quantification of the number of PDX1+ buds per main body (**c**) using the *PDX1* reporter (HUES4 PDXeG) line. n = 4 independent differentiations. Scale bar, 100 µm. (**d**) Quantification of PDX1+ bud formation efficiency in the indicated stem cell lines tested in this study. n = 3-8 independent differentiations. (**e-g**) Representative experiment showing INS+ bud formation in 96-well assays (**e-f**), and quantification of the number of INS+ islet buds per main body (**g**) using the INS reporter (Mel1 *INS*$^{GFP/W}$) line. n = 4 independent differentiations. Scale bar, 100 µm. (**h**) Quantification of INS+ islet bud formation efficiency in the indicated stem cell lines tested in this study. n = 3-8 independent differentiations. Budding differentiation was induced by 100 ng/mL GDF8 plus 1-1.5 µM CHIR99021 in Mel1 *INS*$^{GFP/W}$, HUES4 PDXeG and HUES8 lines, 100 ng/mL GDF8 plus 0.2-0.5 µM CHIR99021 in H1 line, and 100 ng/mL GDF8 plus 0.5-1 µM CHIR99021 in GCaMP hiPSC line for Stage 1 Day 1. Created with BioRender.com released under a Creative Commons Attribution-NonCommercial-NoDerivs 4.0 International license (**a** and **e**).

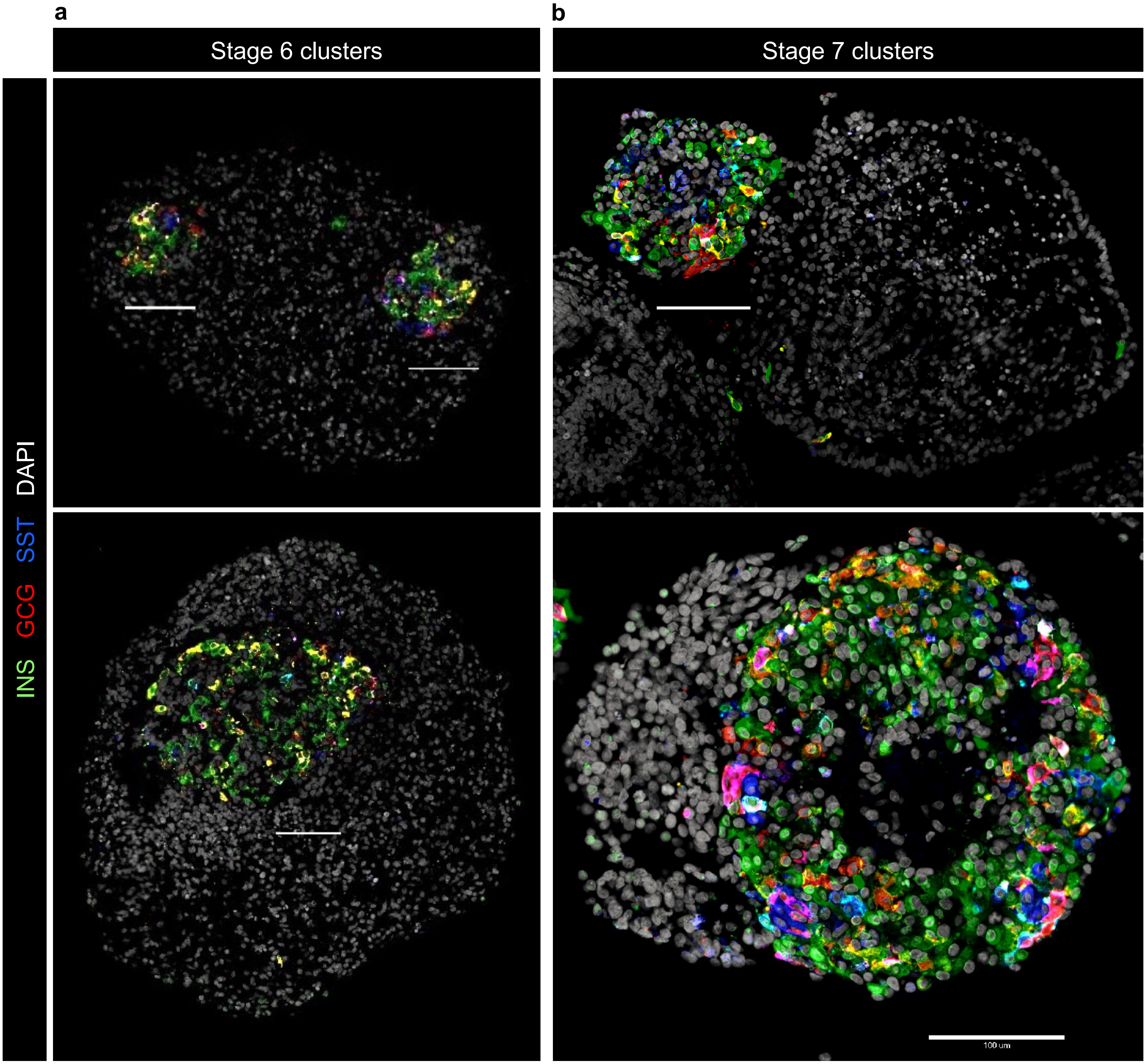

Supplementary Fig. 6: Representative images showing the whole clusters with both islet buds and main bodies.
(**a-b**) Representative section staining images of budding-type Stage 6-7 whole clusters stained with major islet cell types (INS, GCG and SST). Of note, islet cell types were highly enriched in bud structures and rarely seen in main bodies. Scale bars, 100 µm. Budding differentiation was induced by 100 ng/mL GDF8 plus 0.5 µM CHIR99021 using the H1 line.

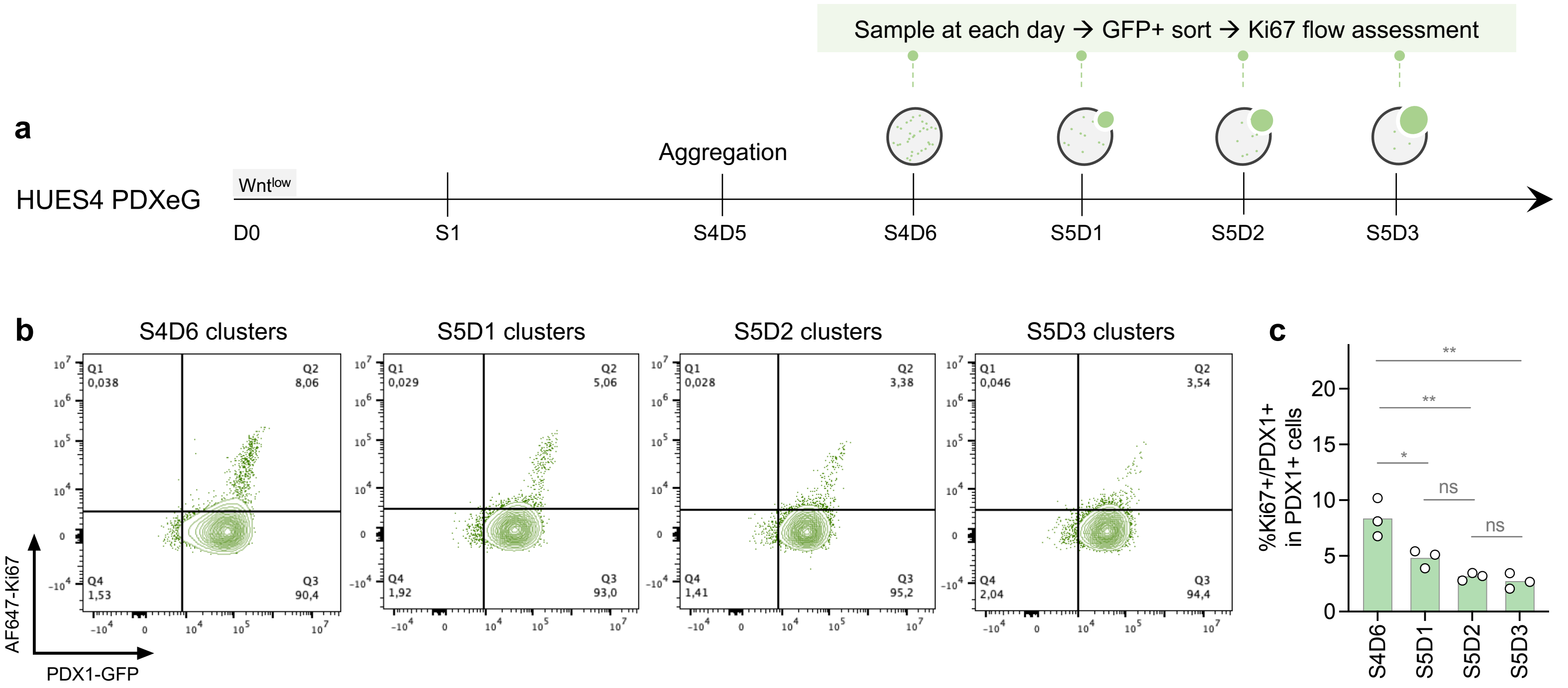

Supplementary Fig. 7: Assessment of PDX1+ cell proliferation rate during budding process.

(**a**) Experimental design for assessing proliferative PDX1+ cells during budding process. Created with BioRender.com released under a Creative Commons Attribution-NonCommercial-NoDerivs 4.0 International license. (**b-c**) Representative flow cytometry plots (**b**) and quantification (**c**) of Stages 4-5 clusters examined by PDX1 and proliferative marker Ki67. n = 3 independent differentiations, ns, not significant, *p < 0.05, **p < 0.01, one-way ANOVA with Dunnett test for multiple comparisons to Stage 4 clusters and Tukey test for comparisons between groups. Budding differentiation was induced by 100 ng/mL GDF8 plus 1-1.5 µM CHIR99021 for Stage 1 Day 1 using HUES4 PDXeG line.

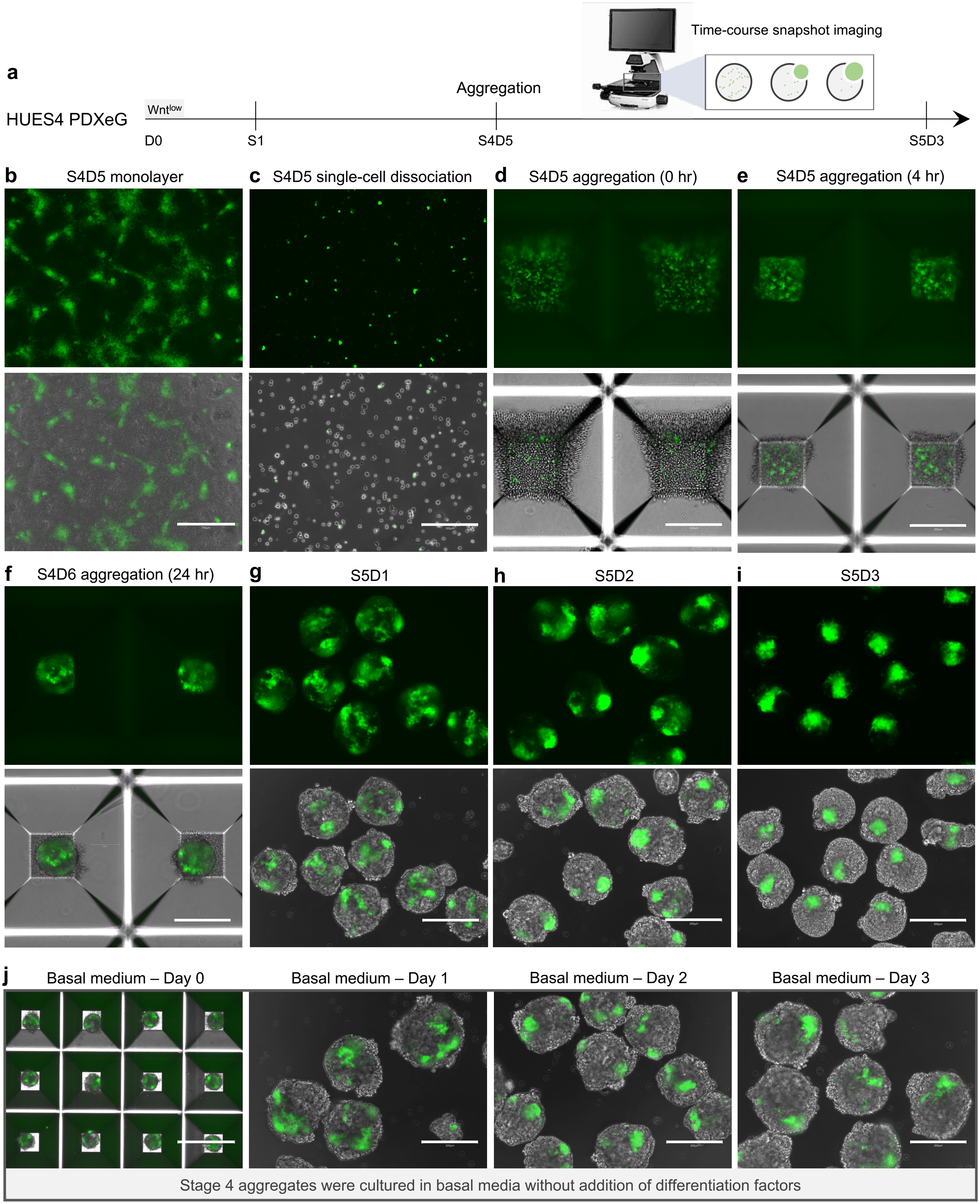

Supplementary Fig. 8: Spontaneous clustering of PDX1+ cells in both planar and suspension cultures.

(**a**) Schematic of budding-type differentiations with the HUES4 PDXeG line for live imaging of PDX1+ cells. Created with BioRender.com released under a Creative Commons Attribution-NonCommercial-NoDerivs 4.0 International license. (**b-i**) Representative images showing the distribution patterns of PDX1+ cells (indicated by PDXeG) at indicated time points. Specifically, PDX1+ cells tended to form clustered structure under planar culture (**b**). Although PDX1+ cells were completely dispersed by dissociation procedure (**c**), after 3D aggregation (**d-f**) they spontaneously coalesced together forming PDX1+ cell-enriched bud niche in aggregates under suspension culture (**g-i**). Scale bars, 300 µm. (**j**) Stage 4 aggregates derived from HUES4 PDXeG cells were cultured in basal medium without inducing factors. The self-aggregation of PDX1+ cells was still observed, indicating a spontaneous clustering process independent of differentiation additives. Scale bar, 750 µm applied to image of Day 0. Scale bars, 300 µm applied to images of Day 1-3. Budding differentiation was induced by 100 ng/mL GDF8 plus 1-1.5 µM CHIR99021 for Stage 1 Day 1 using the HUES4 PDXeG line.

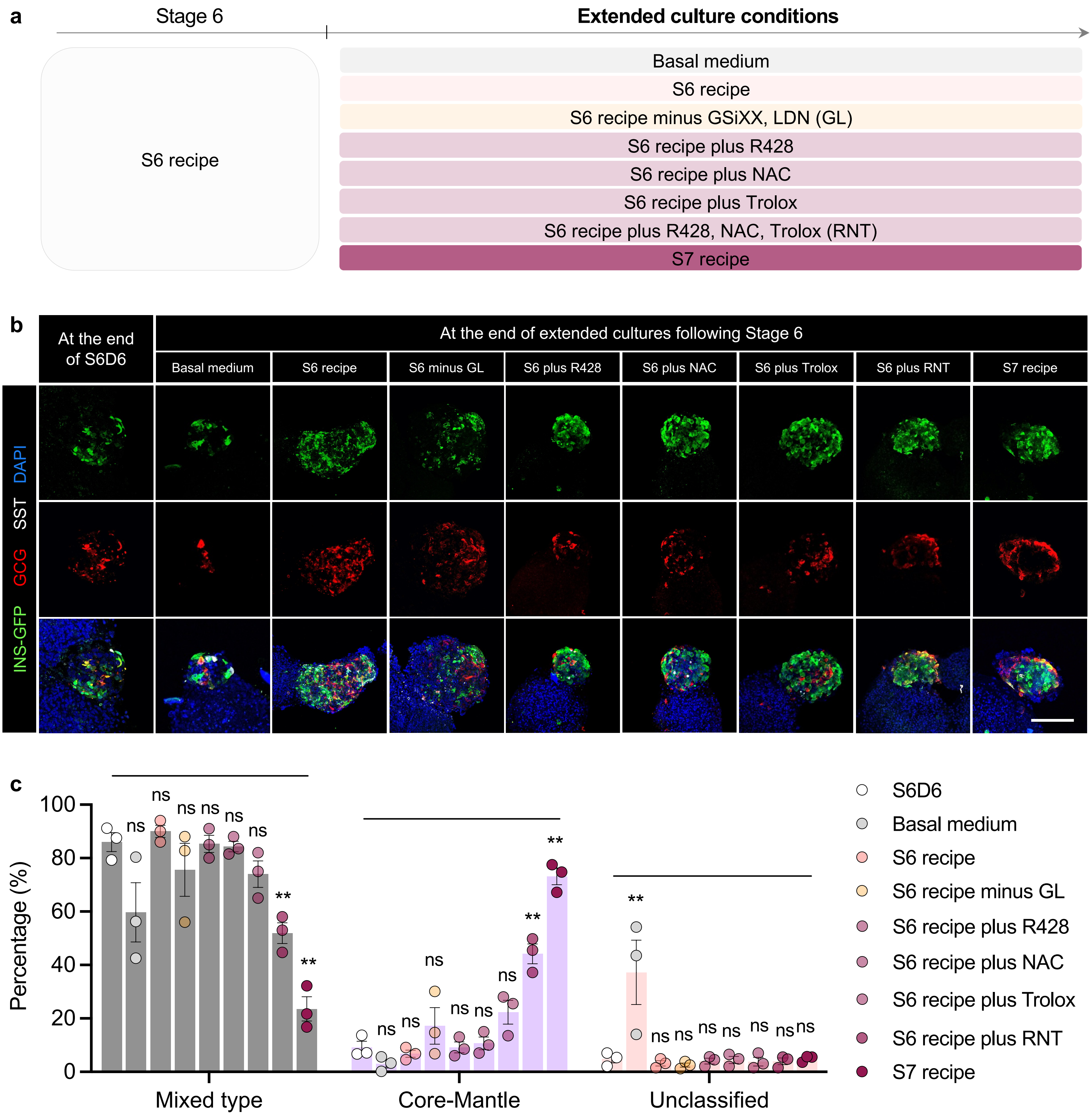

Supplementary Fig. 9: The process of inducing differentiation is required for islet cell rearrangement.

(**a**) Study design for testing the effects of stage-specific additives on islet bud cytoarchitecture in extended cultures following Stage 6. (**b**) Representative images of clusters stained for major islet cell types (INS, GCG, SST). INS expression was indicated by INS-GFP in clusters derived from Mel1 *INS*$^{GFP/W}$ hESCs. Scale bar, 100 µm. (**c**) Quantification showing the percentage of different cytoarchitectural types adopted by clusters that were treated with different conditions as outlined in A. n = 15-20 clusters for each from 3 independent differentiations. One-way ANOVA with Dunnett test (**p < 0.01) for multiple comparisons to Stage 6 Day 6 (S6D6) buds. Budding differentiation was induced by 100 ng/mL GDF8 plus 1-1.5 µM CHIR99021 for Stage 1 Day 1 using the Mel1 *INS*$^{GFP/W}$ line.

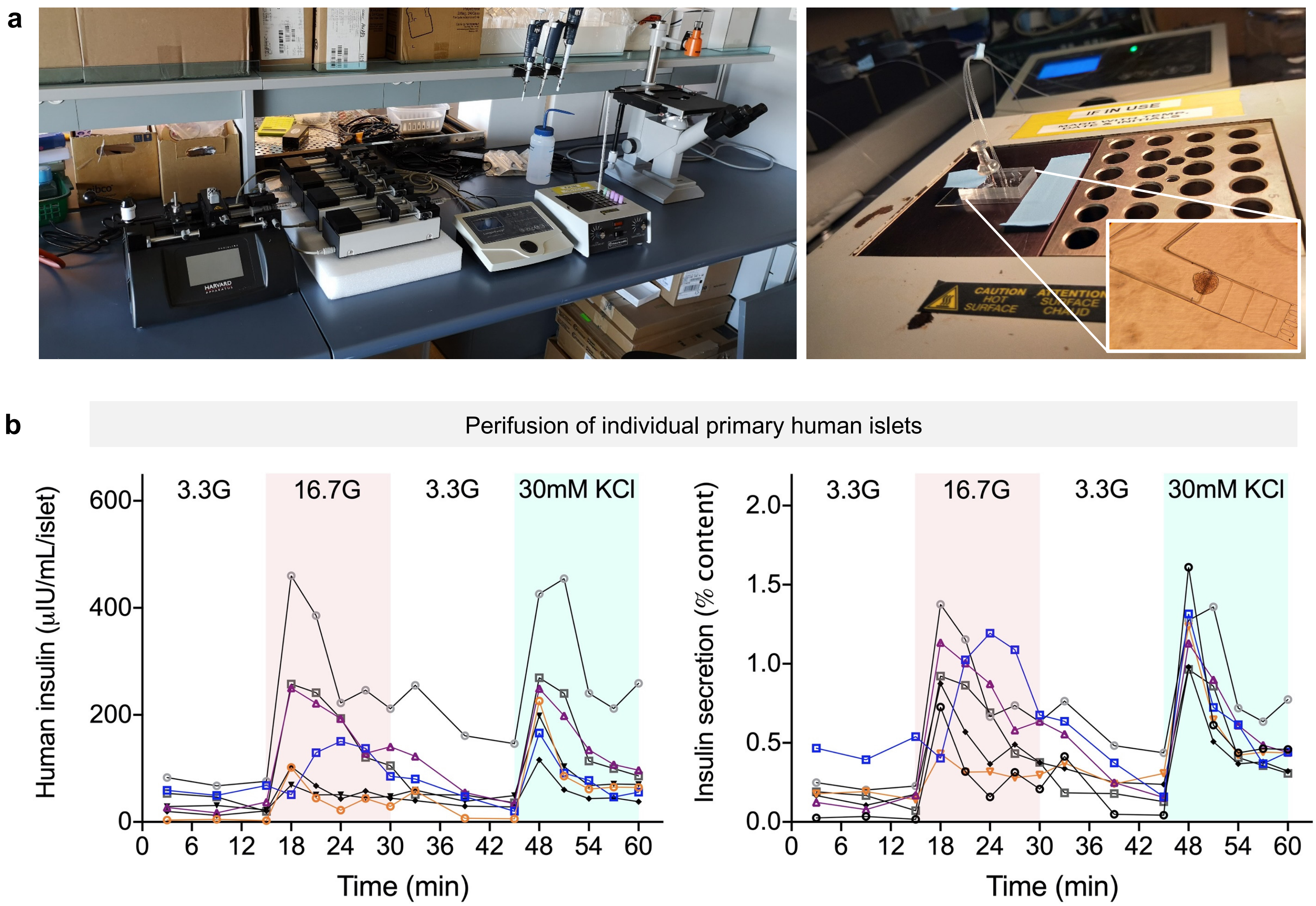

Supplementary Fig. 10: Perifusion of single primary human islet cluster and detection of dynamic insulin secretion in a microfluidic chip system.
(**a**) Microfluidic chip system to perfuse single islet clusters for measuring dynamic insulin secretion, with temperature-controlled reaction stage and program-controlled syringe pump. (**b**) Representative traces (left panel, raw values; right panel, normalized to total insulin content) showing detection of robust and dynamic insulin secretion from single primary human islet. n = 7 islets from 3 independent batches of preparations.

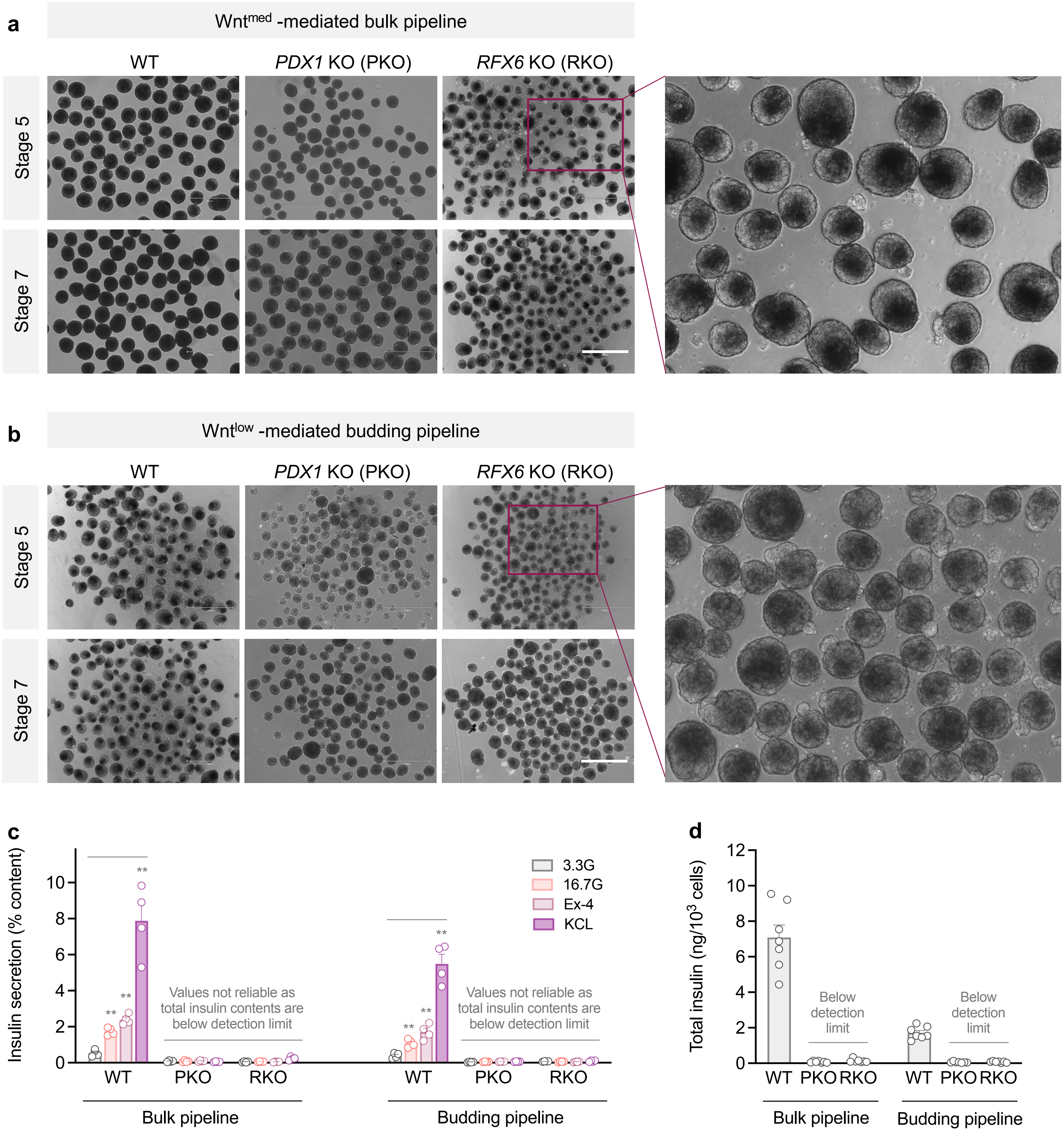

Supplementary Fig. 11: Additional morphological and functional phenotypes in *PDX1* KO and *RFX6* KO hESC cultures.
(**a-b**) Representative phase-contrast images of Stage 5 and Stage 7 clusters derived from parental WT, *PDX1* KO and *RFX6* KO hESC lines showing morphological differences. Bulk- and budding-type differentiation pipelines were applied. Of note, cell budding was not found in *PDX1* KO hESCs regardless of differentiation pipelines; surprisingly, *RFX6* KO hESCs showed typical budding morphogenesis under a bulk differentiation pipeline. Scale bars, 750 µm. (**c-d**) Static GSIS assays showing insulin secretion (**c**) and total insulin content (**d**) from Stage 7 clusters derived from WT, *PDX1* KO and *RFX6* KO hESCs. Values of measurement under detection limit of the ELISA kit are shown but not reliable to include for quantification. n = 4 independent batches of differentiations, one-way ANOVA with Dunnett test (ns, not significant, *p < 0.05, **p < 0.01) for multiple comparisons to indicated common controls. Budding differentiation was induced by 100 ng/mL GDF8 plus 1.5 µM CHIR99021 for Stage 1 Day 1 using the HUES8 WT, *PDX1* KO and *RFX6* KO lines.

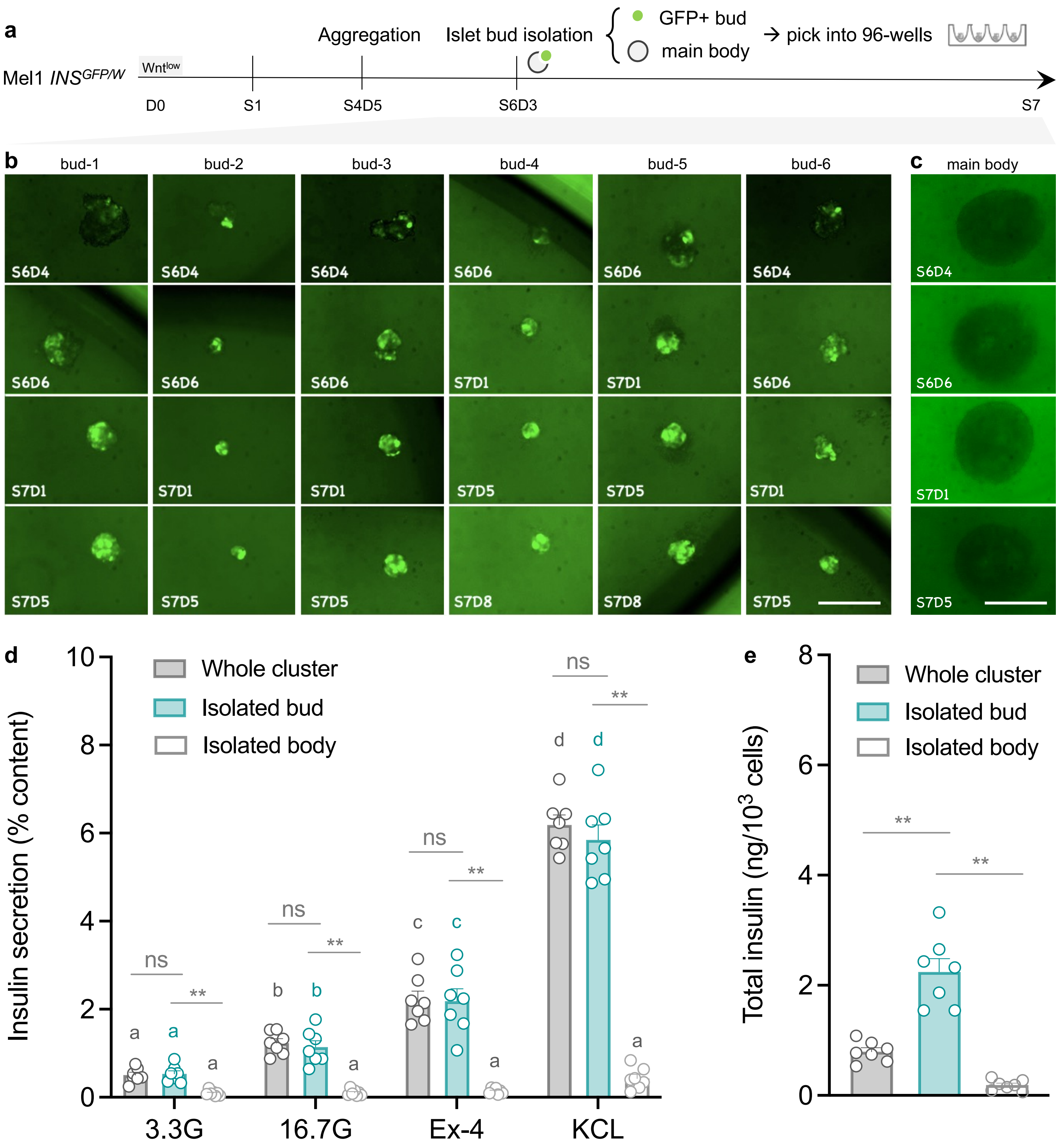

Supplementary Fig. 12: Purified islet buds continue to differentiate after separation from main bodies.
(**a-c**) Schematic (**a**) and representative images (**b-c**) of budding-type differentiations with Mel1 *INS*$^{GFP/W}$ line for live cell tracking of individual GFP+ buds and GFP- bodies at indicated stages. Scale bars, 200 µm. Created with BioRender.com released under a Creative Commons Attribution-NonCommercial-NoDerivs 4.0 International license in (**a**). (**d-e**) Static GSIS assays showing insulin secretion (**d**) and total insulin (**e**) from Stage 7 whole clusters, isolated buds and isolated main bodies. n = 7 independent differentiations, one-way ANOVA with Dunnett test (ns, not significant, $^{**}p < 0.01$) for multiple comparisons to isolated buds, one-way ANOVA with Tukey test (with different letters) for multiple comparisons between secretagogues within same group. Budding differentiation was induced by 100 ng/mL GDF8 plus 1-1.5 µM CHIR99021 for Stage 1 Day 1 using the Mel1 *INS*$^{GFP/W}$ line.

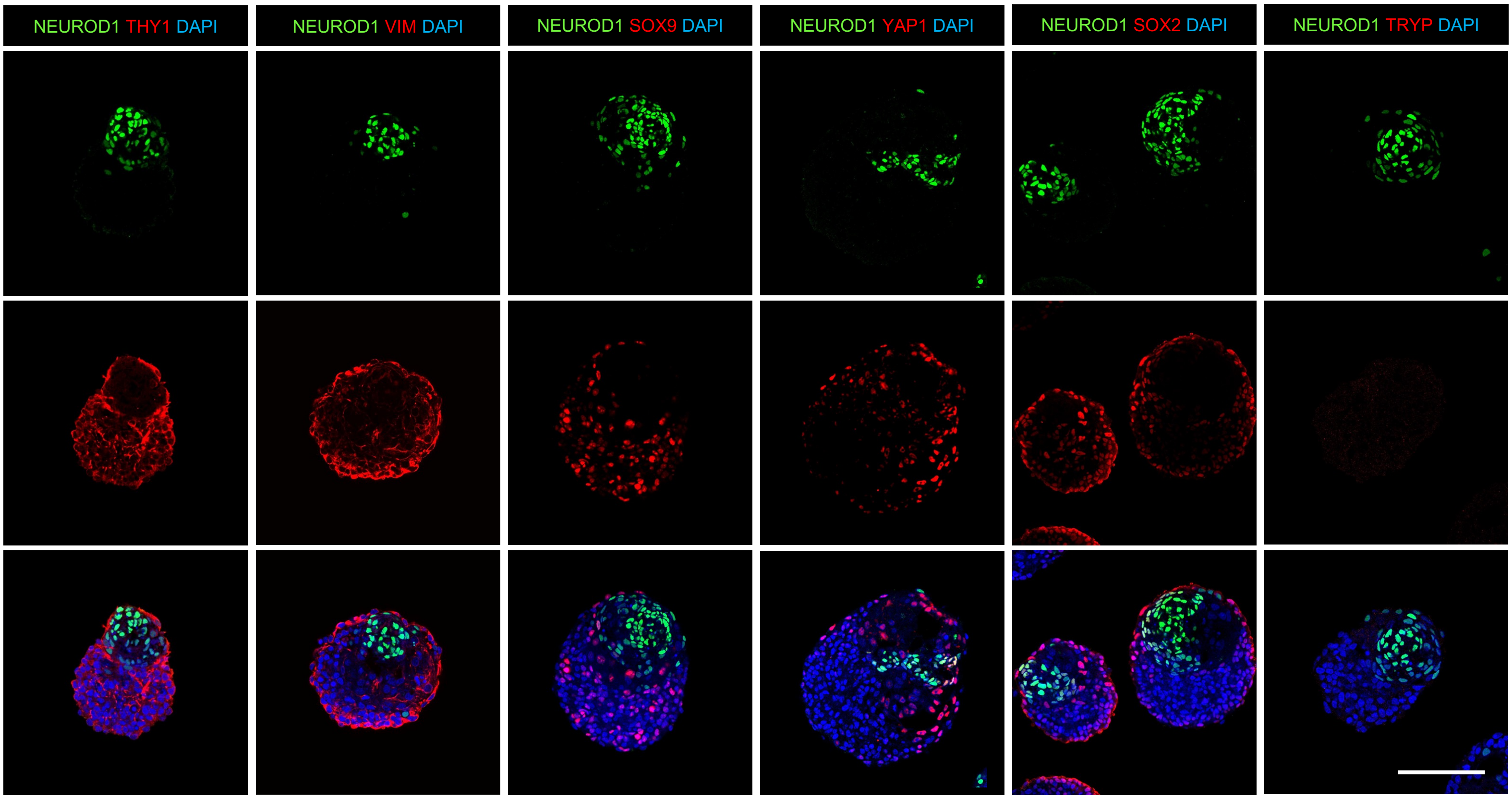

Supplementary Fig. 13: Additional characterization of main body cells generated by budding-type differentiation.

Representative immunostaining images of Stage 5 clusters stained for NEUROD1 and various targets as informed by RNA-seq and qPCR analysis. Budding differentiation was induced by 100 ng/mL GDF8 plus 0.2-0.5 µM CHIR99021 for Stage 1 Day 1 using the H1 line.

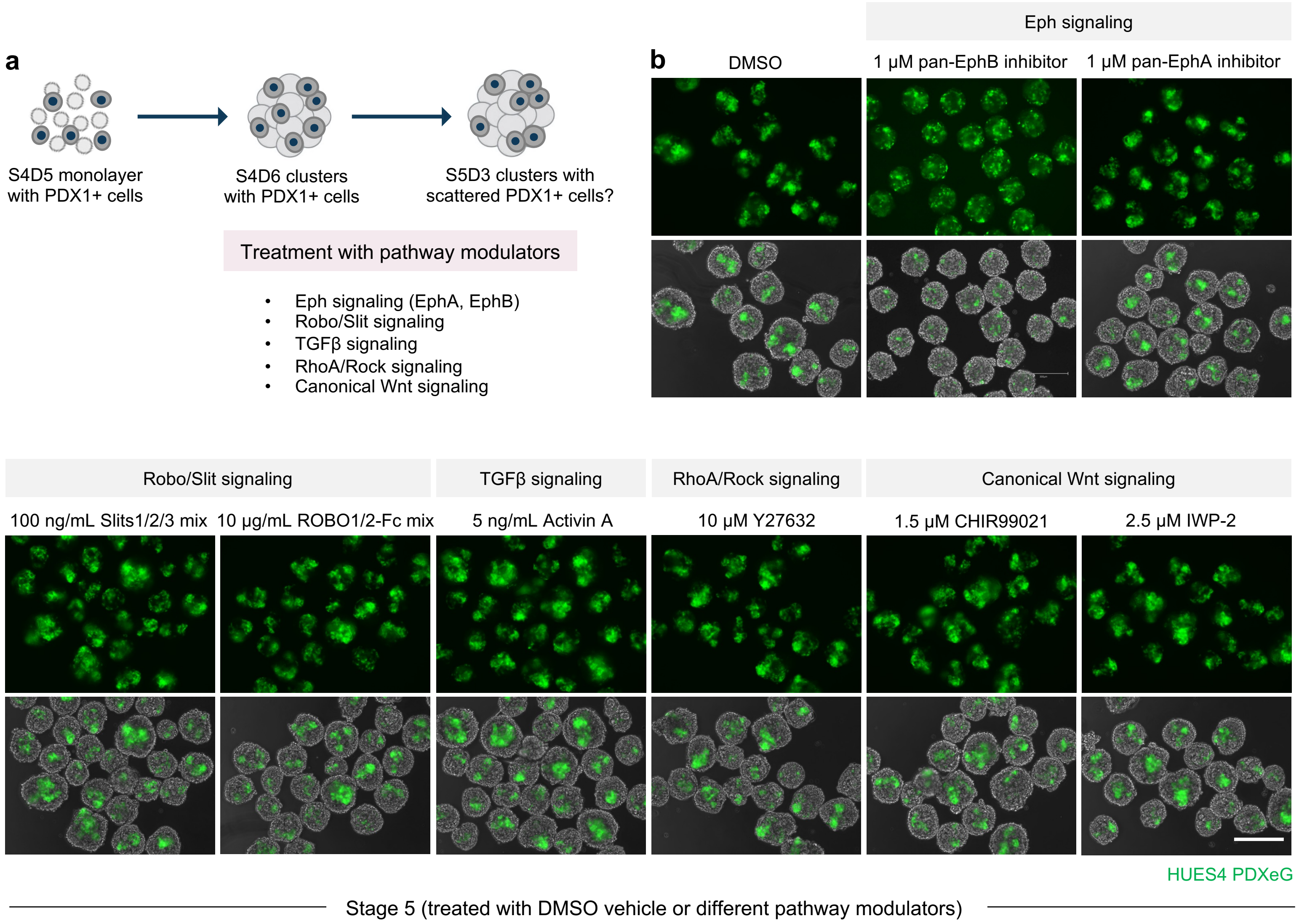

Supplementary Fig. 14: Screening of different signaling pathways on PDX1+ cell clustering.
(**a**) Experimental design for initial screen of different signaling pathways on PDX1+ cell clustering. Created with BioRender.com released under a Creative Commons Attribution-NonCommercial-NoDerivs 4.0 International license. (**b**) Representative images of Stage 5 clusters that were treated with DMSO vehicle and indicated inhibitors or agonists at budding stage. Note, pathway modulators were supplemented to Stage 5 complete medium, except for Activin A. Activin A was supplemented to Stage 5-minus medium (i.e., complete medium minus TGFβ inhibitor ALK5i). HUES4 PDXeG line was used for differentiation and for imaging PDX1+ cells in living cultures. Scale bar, 300 μm. Budding differentiation was induced by 100 ng/mL GDF8 plus 1.5 μM CHIR99021 for Stage 1 Day 1 using the HUES4 PDXeG line.

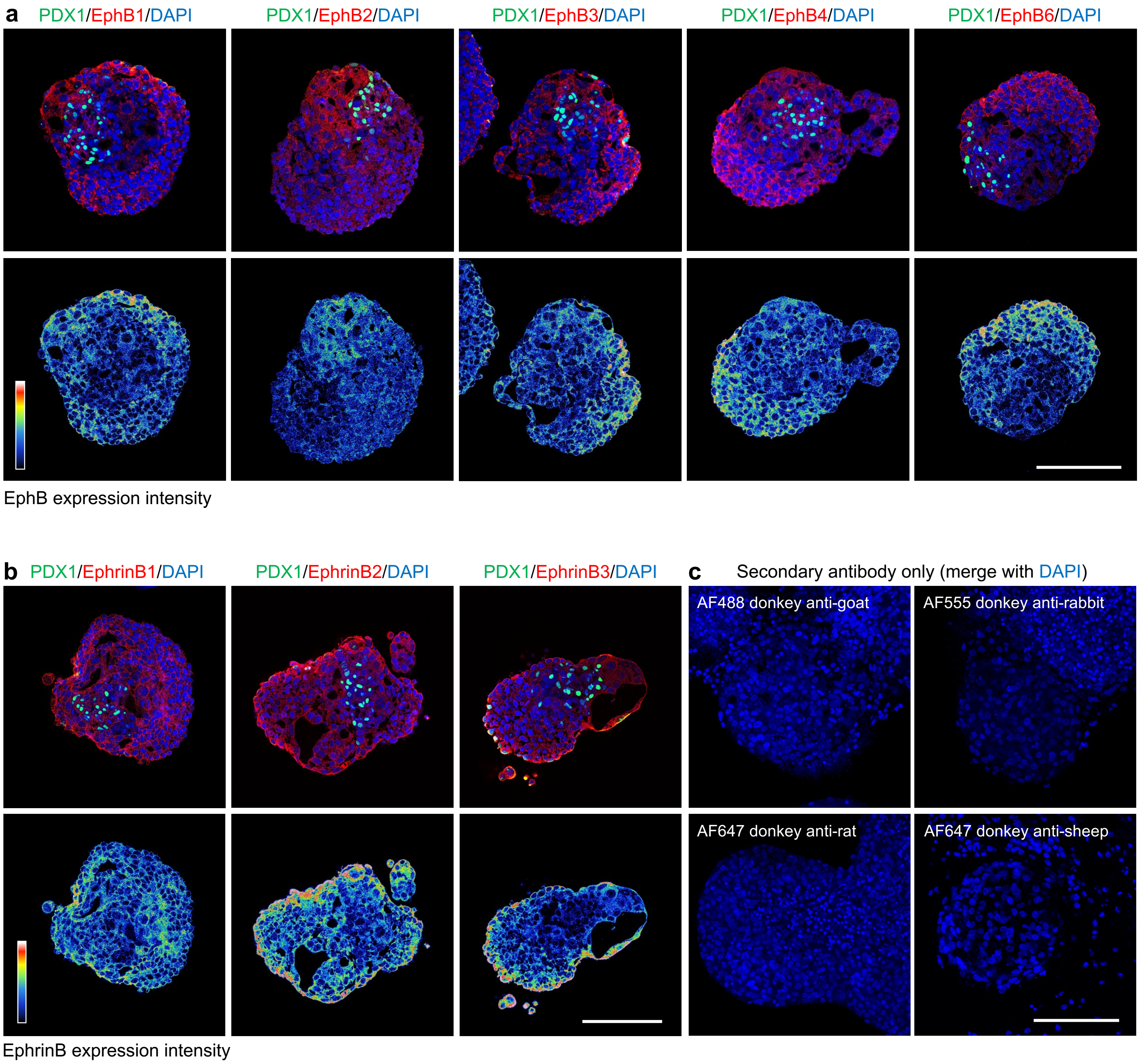

Supplementary Fig. 15: Differential expression of EphB/EphrinB in endocrine buds and main bodies.
(**a**-**b**) Top panels show representative images of Stage 5 clusters stained for PDX1 and EphB or EphrinB. Bottom panels show the spatial expression intensities of EphB or EphrinB with spectrum pseudo-colored. Nuclei were counterstained with DAPI (blue). Scale bars, 100 µm. (**c**) Representative images of staining controls with secondary antibody only. Scale bar, 100 µm. Budding differentiation was induced by 100 ng/mL GDF8 plus 0.5 µM CHIR99021 for Stage 1 Day 1 using the H1 line.

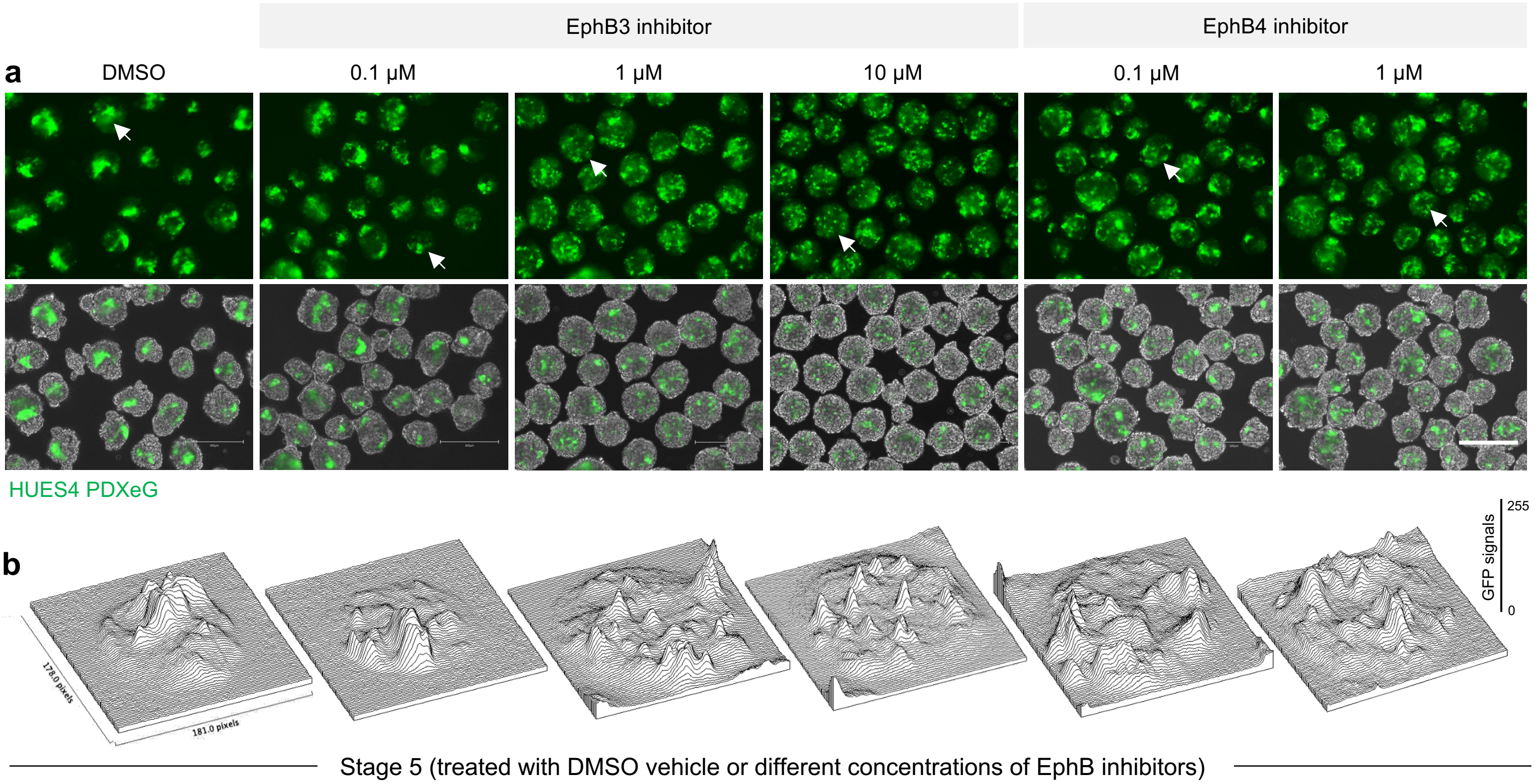

Supplementary Fig. 16: Inhibition of EphB3/4 signaling interrupts PDX1+ cell clustering.

(**a**) Representative images of Stage 5 clusters that were treated with DMSO vehicle, selective EphB3 or EphB4 inhibitors at budding stage. Dose responses of EphB3 and EphB4 inhibitors were tested. HUES4 PDXeG line was used for differentiation and for imaging PDX1+ cells in living cultures. Scale bar, 300 µm. (**b**) Surface plot analysis showing PDX1+ cell distribution patterns in representative Stage 5 clusters as indicated by arrowheads in **a**. Budding differentiation was induced by 100 ng/mL GDF8 plus 1.5 µM CHIR99021 for Stage 1 Day 1 using the HUES4 PDXeG line.

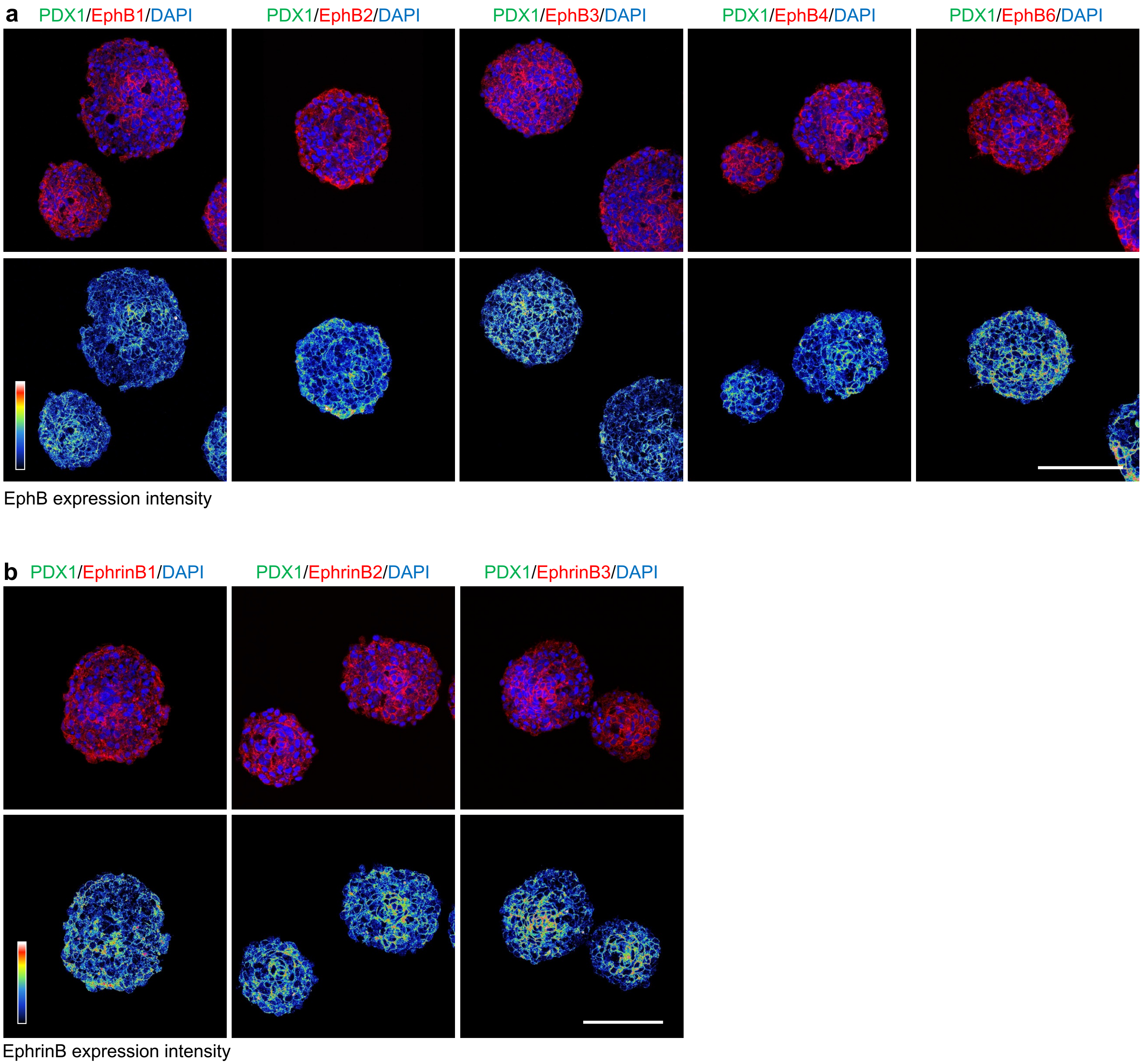

Supplementary Fig. 17: Examination of EphB/EphrinB expression in Stage 5 clusters derived from *PDX1* KO hESCs.
(**a-b**) Top panels show representative images of Stage 5 clusters stained for PDX1 and EphB or EphrinB. Bottom panels show the spatial expression intensities of EphB or EphrinB with spectrum pseudo-colored. Nuclei were counterstained with DAPI (blue). Scale bars, 100 μm. Budding differentiation was induced by 100 ng/mL GDF8 plus 1.5 μM CHIR99021 for Stage 1 Day 1 using the *PDX1* KO hESC line.

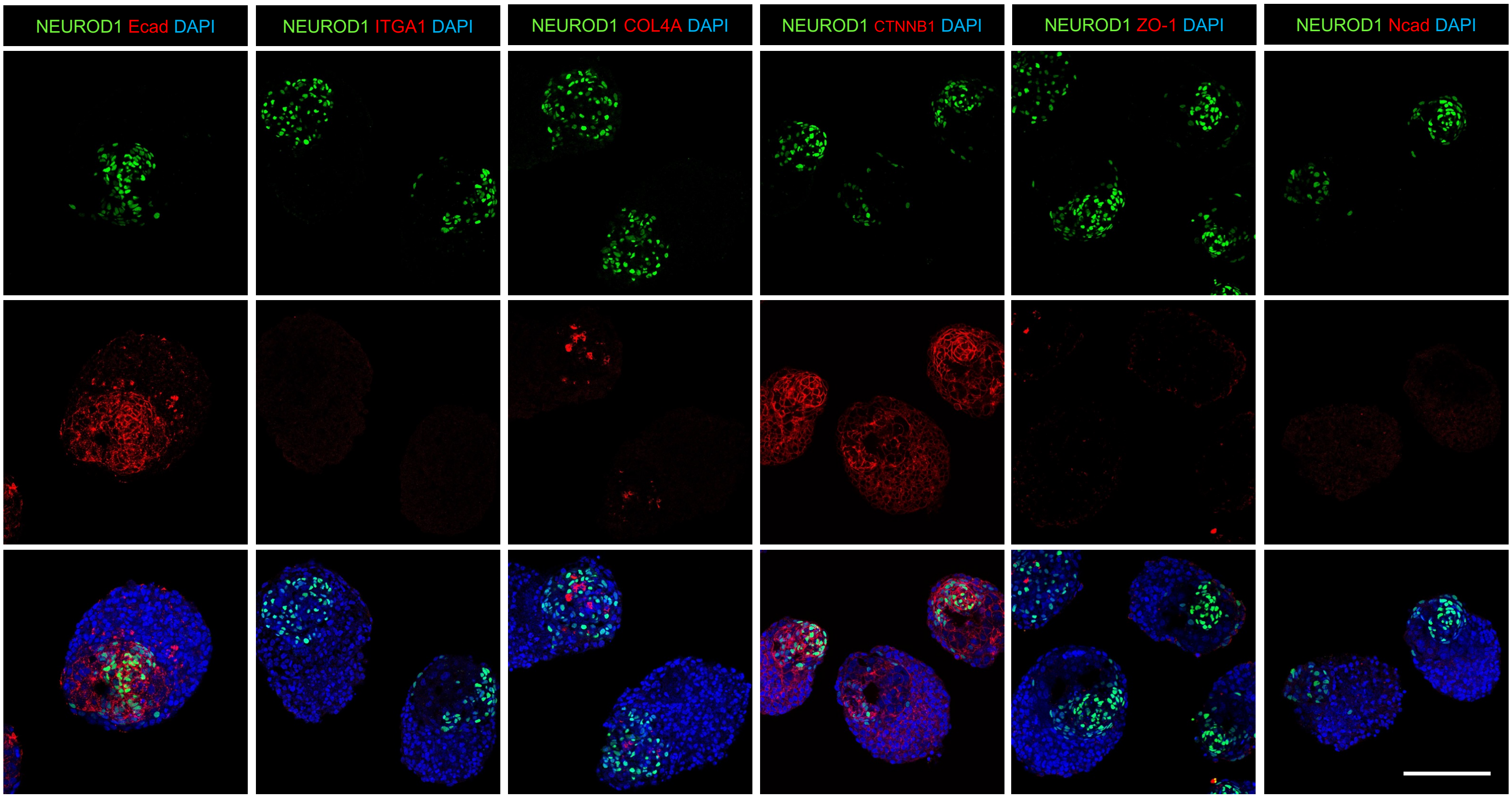

Supplementary Fig. 18: Staining of adhesion molecules, EMT markers and cell polarity markers.
Representative images of Stage 5 clusters stained for NEUROD1 and various adhesion molecules, EMT markers and cell polarity markers, including E-Cadherin (Ecad), integrin (ITGA1), beta-catenin (CTNNB1), collagen (COL4A1/A2), ZO-1 (marking tight junctions) and N-Cadherin (Ncad). Nuclei were counterstained with DAPI (blue). Scale bars, 100 µm. Budding differentiation was induced by 100 ng/mL GDF8 plus 0.2-0.5 µM CHIR99021 for Stage 1 Day 1 using the H1 line.

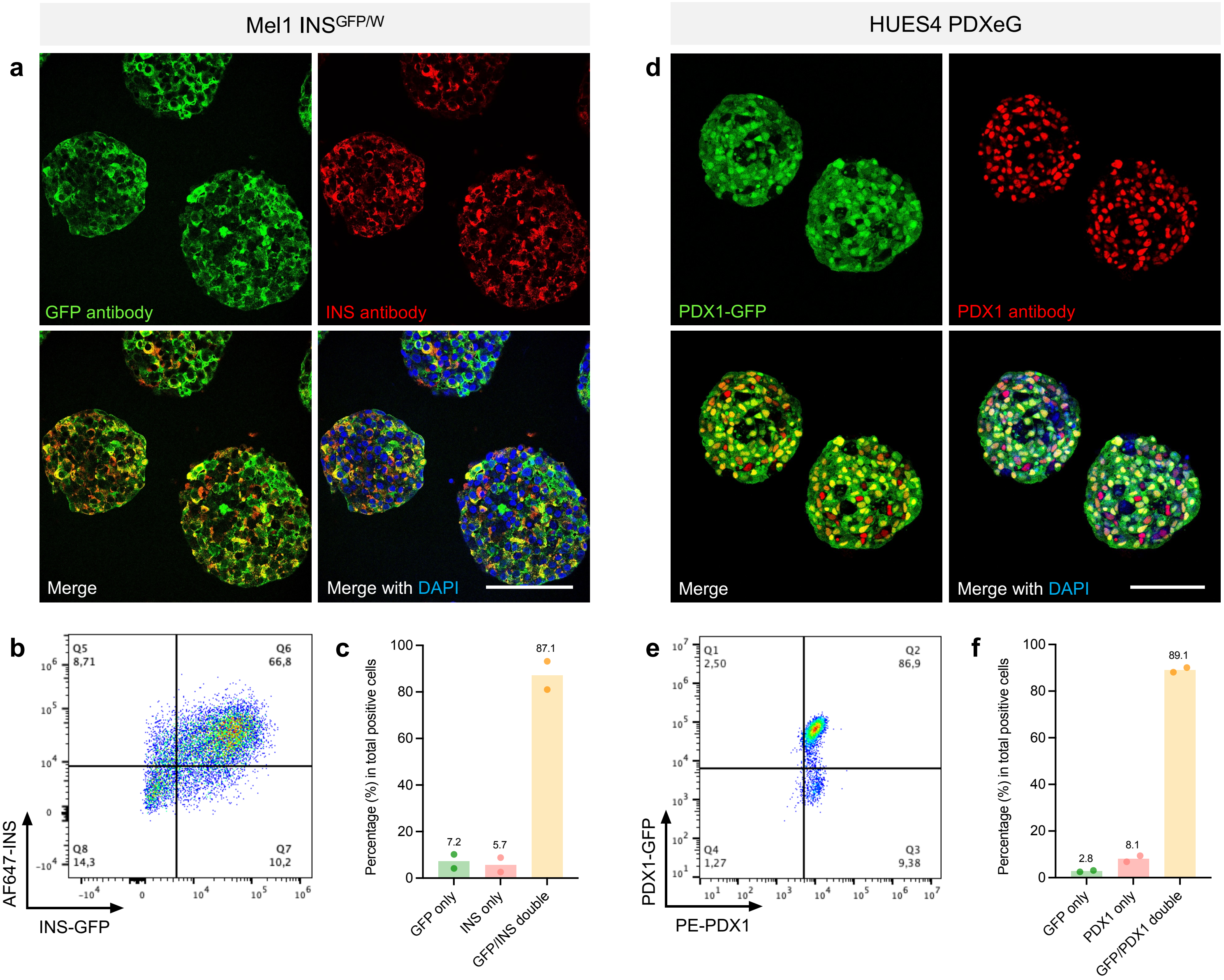

Supplementary Fig. 19: Validation of Mel1 *INS$^{GFP/W}$* and HUES4 PDXeG reporter lines.

(**a**) Representative section staining images of bulk-type Stage 7 islet clusters derived from Mel1 *INS$^{GFP/W}$* line stained with GFP antibody (green) and insulin antibody (red). Scale bar, 100 µm. (**b-c**) Representative flow plot (**b**) and quantification (**c**) of bulk-type Stage 7 islet clusters derived from Mel1 *INS$^{GFP/W}$* line examined for endogenous INS-GFP signals and insulin signals by insulin antibody. n = 2 independent differentiations. (**d**) Representative whole-mount staining images of bulk-type Stage 4 pancreatic progenitors derived from HUES4 PDXeG line examined for endogenous PDX1-GFP signals (green) and PDX1 signals by PDX1 antibody (red). Scale bar, 100 µm. (**e-f**) Representative flow plot (**e**) and quantification (**f**) of bulk-type Stage 4 pancreatic progenitors derived from HUES4 PDXeG line examined for endogenous PDX1-GFP signals and PDX1 signals by PDX1 antibody. n = 2 independent differentiations. Bulk-type differentiation was induced by 100 ng/mL GDF8 plus 3 µM CHIR99021 in both Mel1 *INS$^{GFP/W}$* and HUES4 PDXeG lines.

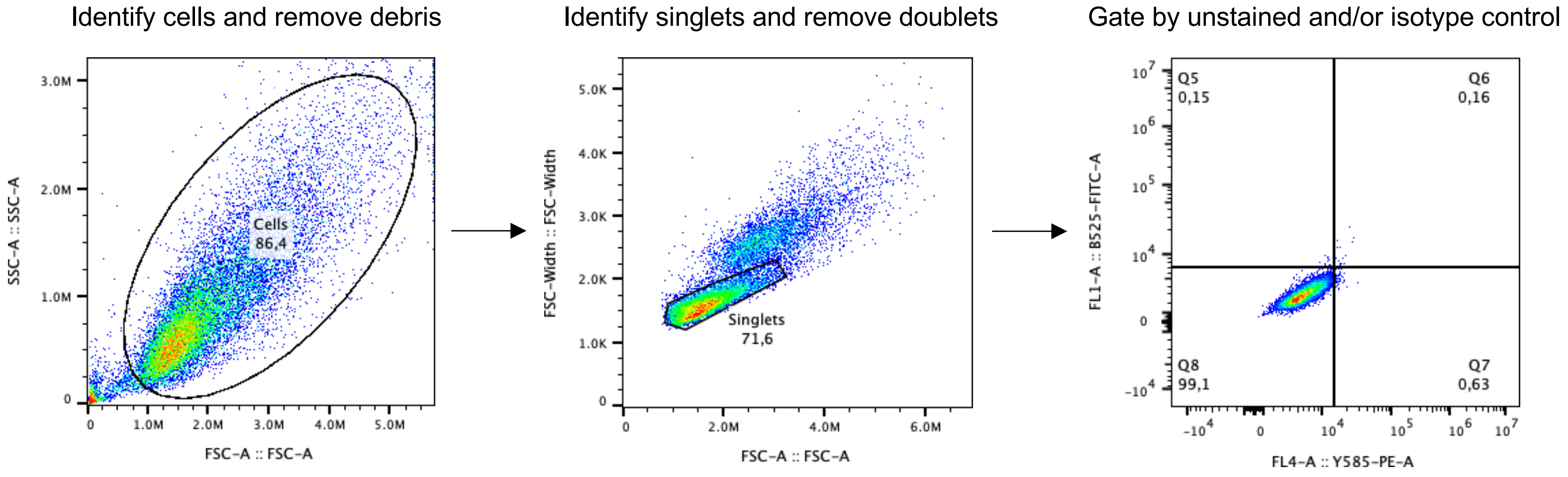

Supplementary Fig. 20: An example of gating strategy for flow cytometry experiments in this study.

All captured events were first plotted by forward scatter area (FSC-A) and side scatter area (SSC-A). In this plot, debris were removed and cells were identified. Next, singlets were identified by gating based on the plots by forward scatter area (FSC-A) and forward scatter width (FSC-W). Finally, flow cytometry plots were gated by unstained and/or isotype controls on fluorophore channels.

Supplementary Table 1: Comparison of differentiation process and summary of Wnt^{low}-mediated budding-type and Wnt^{med}-mediated bulk-type systems.

	**Budding-type differentiation system**	**Bulk-type differentiation system**
Endoderm specification	• Endoderm specification under Wnt^{low} condition<br>• Induction of 70%-85% FOXA2+/SOX17+ endoderm cells at S1	• Endoderm specification under Wnt^{med} condition<br>• Induction of 90%-99% FOXA2+/SOX17+ endoderm cells at S1
Pancreas specification	• PDX1 starts from S3<br>• Delayed activation of NKX6.1<br>• Induction of 20%-40% PDX1+ cells at S3-S4<br>• Rare presence of NKX6.1+ cells (< 5%) at S4<br>• Low levels of pro-endocrine cell transcripts (NGN3 and NEUROD1) at S4	• PDX1 starts from S3; NKX6.1 starts from S4<br>• Induction of 85%-95% PDX1+ cells at S3-S4<br>• Induction of 75%-90% PDX1+/NKX6.1+ pancreatic progenitor cells at S4<br>• Medium levels of pro-endocrine cell transcripts (NGN3 and NEUROD1) at S4
Pancreatic bud formation	• PDX1+ cell migration and clustering within a heterogeneous cellular environment<br>• NKX6.1 expression increases in PDX1+ buds	• PDX1+ bud formation is not observed<br>• PDX1 and NKX6.1 express throughout whole pancreatic progenitor clusters
Endocrine specification	• Endocrine specification in buds only<br>• Buds further protrude from main bodies while keeping the connection<br>• CHGA and ISL1 start from S5 in buds<br>• Endocrine commitment to all major islet cell types (INS, GCG, SST, PPY, GHRL)<br>• Presence of SLC18A1+ enterochromaffin cells<br>• Presence of ~ 30% INS+/GCG+ bi-hormonal cells in S6 buds<br>• Increase in proportions of INS+ and GCG+ monohormonal cells in S7 buds	• Endocrine specification in a certain population of progenitor cells<br>• Bud formation is not observed throughout later endocrine specification stages<br>• CHGA and ISL1 start from S5<br>• Endocrine commitment to all major islet cell types (INS, GCG, SST, PPY, GHRL)<br>• Presence of SLC18A1+ enterochromaffin cells<br>• Presence of ~ 10% INS+/GCG+ bi-hormonal cells in S6-S7 islet clusters<br>• Increase in proportions of GCG+ cells at S7
Islet morphogenesis	• Cytoarchitectural change occurs during S6-S7<br>• Change from an intermingled organization of islet cell types in S6 buds to a core-mantle structure with INS+ cells in the center and GCG+ cells in the periphery in S7 buds	• Islet clusters adopt an intermingled organization of major islet cell types throughout S6-S7<br>• Local enrichment of INS+ cells or GCG+ cells may be seen in some S7 islet clusters
Islet function	• ~ 4.8 ng insulin on average per 10^3 INS+ cells in S7 islet buds<br>• S7 islet buds show ~ 1.7 stimulation index in response to high glucose	• ~ 8.1 ng insulin on average per 10^3 INS+ cells in S7 islet clusters<br>• S7 islet clusters show ~ 2.4 stimulation index in response to high glucose
**Summary of system utility and advantages**	• Serves as an islet developmental model with human cells<br>• Captures multiple morphological events during human islet development<br>• Provides both morphological and transcriptional insights into human islet development<br>• Creates an asynchronous differentiation system with heterogeneous cell-cell interactions<br>• Offers opportunities to study pancreatic cell sorting, tissue segregation, islet morphogenesis and pancreas disease modelling	• Serves as a robust and relatively uniform differentiation system for in vitro production of insulin-producing islet clusters<br>• Promises as an unlimited transplantable source for both pre-clinical and clinical stem cell-based replacement therapy<br>• Provides important transcriptional insights into human islet development<br>• Offers opportunities to study pancreatic and endocrine lineage development as well as pancreas disease modelling

Supplementary Table 2: Key reagents and resources used in this study.

REAGENT or RESOURCE	SOURCE	IDENTIFIER
**Antibodies**		
Rabbit polyclonal anti-PDX1	Abcam	Cat# ab47267
Mouse monoclonal anti-NKX6.1	DSHB	Cat# F55A12
Goat polyclonal anti-NEUROD1	R&D Systems	Cat# AF2746
Sheep polyclonal anti-NGN3	R&D Systems	Cat# AF3444
Mouse monoclonal anti-NGN3	DSHB	Cat# F25A1B3
Rabbit polyclonal anti-Synaptophysin	Novus Biologicals	Cat# NB120-16659
Mouse monoclonal anti-Chromogranin A	DAKO	Cat# M0869
Guinea pig polyclonal anti-Insulin	Abcam	Cat# ab195956
Mouse monoclonal anti-Glucagon	Sigma-Aldrich	Cat# G2654
Rabbit polyclonal anti-Somatostatin	Sigma-Aldrich	Cat# HPA019472
Goat polyclonal anti-Pancreatic polypeptide	R&D Systems	Cat# AF6297
Goat polyclonal anti-PDX1	R&D Systems	Cat# AF2419
Rabbit polyclonal anti-EphB1	Thermo Fisher Scientific	Cat# PA5-14604
Rat monoclonal anti-EphB2	R&D Systems	Cat# MAB467
Rabbit polyclonal anti-EphB3	Abcam	Cat# ab133742
Goat polyclonal anti-EphB4	R&D Systems	Cat# AF3038
Sheep polyclonal anti-EphB6	R&D Systems	Cat# AF3384
Rabbit polyclonal anti-EphrinB1	Sigma-Aldrich	Cat# HPA067188
Goat polyclonal anti-EphrinB2	R&D Systems	Cat# AF496
Goat polyclonal anti-EphrinB3	R&D Systems	Cat# AF395
Rabbit polyclonal anti-SOX9	Millipore	Cat# ab5535
Mouse monoclonal anti-THY1	Abcam	Cat# ab181469
Mouse monoclonal anti-SOX2	Abcam	Cat# 79351
Rabbit polyclonal anti-YAP1	Cell Signaling Technology	Cat# 8418S
Mouse monoclonal anti-ITGA1	BD Biosciences	Cat# 559594
Rabbit polyclonal anti-E-Cadherin	Cell Signaling Technology	Cat# 3195P
Mouse monoclonal anti-COL4A1/A2	DSHB	Cat# M3F7
Mouse monoclonal anti-ZO-1	Thermo Fisher Scientific	Cat# 61-7300
Rabbit polyclonal anti-beta-catenin (CTNNB1)	Cell Signaling Technology	Cat# 8480S
Rat monoclonal anti-Vimentin (VIM)	R&D Systems	Cat# MAB2105
Rat monoclonal anti-N-Cadherin	Thermo Fisher Scientific	Cat# 132100
Rabbit polyclonal anti-GFP	Santa Cruz	Cat# sc-8334
Alexa Fluor 555 donkey anti-mouse IgG	Thermo Fisher Scientific	Cat# A31570
Alexa Fluor 647 donkey anti-mouse IgG	Thermo Fisher Scientific	Cat# A31571
Alexa Fluor 488 donkey anti-rabbit IgG	Thermo Fisher Scientific	Cat# A21206
Alexa Fluor 555 donkey anti-rabbit IgG	Thermo Fisher Scientific	Cat# A31572
Alexa Fluor 647 donkey anti-rabbit IgG	Thermo Fisher Scientific	Cat# A31573
Alexa Fluor 488 donkey anti-goat IgG	Thermo Fisher Scientific	Cat# A11055
Alexa Fluor 555 donkey anti-goat IgG	Thermo Fisher Scientific	Cat# A21432
Alexa Fluor 647 donkey anti-goat IgG	Thermo Fisher Scientific	Cat# A21447
Alexa Fluor 488 donkey anti-guinea pig IgG	Jackson Immuno Research	Cat# 706-545-148
Alexa Fluor 555 donkey anti-guinea pig IgG	Biotium	Cat# 20276
Alexa Fluor 647 donkey anti-sheep IgG	Thermo Fisher Scientific	Cat# A21448
Alexa Fluor 647 donkey anti-rat IgG	Thermo Fisher Scientific	Cat# A78947

PE mouse anti-PDX1	BD Biosciences	Cat# 562161
Alexa Fluor 647 mouse anti-NKX6.1	BD Biosciences	Cat# 563338
Alexa Fluor 647 mouse anti-NEUROD1	BD Biosciences	Cat# 563566
PE mouse anti-CHGA	BD Biosciences	Cat# 564563
Alexa Fluor 647 mouse anti-Insulin	BD Biosciences	Cat# 565689
PE mouse anti-Glucagon	BD Biosciences	Cat# 565860
Alexa Fluor 488 mouse anti-Somatostatin	BD Biosciences	Cat# 566032
PE mouse IgG1κ, isotype control	BD Biosciences	Cat# 554680
Alexa Fluor 488 mouse IgG2b, κ, isotype control	BD Biosciences	Cat# 558716
Alexa Fluor 647 mouse IgG1κ, isotype control	BD Biosciences	Cat# 557714
**Biological samples**		
Human islets	ADI Islet Core	https://www.epicore.ualberta.ca/IsletCore/
**Chemicals, peptides, and recombinant proteins**		
Matrigel, hESC-qualified	Corning	Cat# 08-774-552
TrypLE Express Enzyme	Thermo Fisher Scientific	Cat# 12604021
Accutase	STEMCELL Technologies	Cat# 07920
Gentle Cell Dissociation Reagent	STEMCELL Technologies	Cat# 07174
Y-27632	STEMCELL Technologies	Cat# 72304
mTeSR1 Complete Kit	STEMCELL Technologies	Cat# 85850
DPBS, without Ca^{2+} and Mg^{2+}	Sigma-Aldrich	Cat# D8537
DMEM/F-12, HEPES	Thermo Fisher Scientific	Cat# 11330-032
MCDB131 medium	Life technologies	Cat# 10372019
CMRL 1066, Supplemented, CIT modification	Corning	Cat# 98-304-CV
Glutamax	Thermo Fisher Scientific	Cat# 35050061
ITS-X	Thermo Fisher Scientific	Cat# 51500056
$NaHCO_3$	Sigma-Aldrich	Cat# S6297
D-glucose	Sigma-Aldrich	Cat# G8769
Fatty acid-free BSA	Proliant	Cat# 68700
GDF-8	PeproTech	Cat# 120-00
Activin A	R&D Systems	Cat# 338-AC
MCX-928	Janssen Internal compound	N/A
CHIR99021	Sigma-Aldrich	Cat# SML1046
mWnt3A	R&D Systems	Cat# 1324-WN
L-ascorbic acid	Sigma-Aldrich	Cat# A4544
FGF-7	R&D Systems	Cat# 251-KG
SANT-1	Sigma-Aldrich	Cat# S4572
Retinoid acid, all-trans	Sigma-Aldrich	Cat# R2625
LDN193189	STEMCELL Technologies	Cat# 72147
TPPB	Tocris	Cat# 5343/1
Triiodothyronine (T3)	Sigma-Aldrich	Cat# T6397
ALK5 inhibitor II	Cayman Chemicals	Cat# 14794
Zinc sulfate	Sigma-Aldrich	Cat# Z0251
Heparin	Sigma-Aldrich	Cat# H3149
Gamma secretase inhibitor XX (GSi XX)	Sigma-Aldrich	Cat# 565789
N-acetyl cysteine (NAC)	Sigma-Aldrich	Cat# A9165
Trolox	Sigma-Aldrich	Cat# 648471
R428	Cayman Chemicals	Cat# 21523

**Critical commercial assays**		
MycoSEQ Mycoplasma Detection Kit	Life technologies	Cat# 4399363
Fixation/Permeabilization Solution Kit	BD Biosciences	Cat# 554714
TaqMan™ Human WNT qPCR Array Plate Kit	Thermo Fisher Scientific	Cat# 4414100
GeneQuery™ Human WNT qPCR Array Plate Kit	ScienCell	Cat# GK026
Human Insulin ELISA Kit	ALPCO	Cat# 80-INSHU-E01.1
Ultralow Attachment Flat Bottom 96-well plate	Corning Costar (VWR)	Cat# CLS3474
Aggrewell™ 400, 24-well plate	STEMCELL Technologies	Cat# 34415
Aggrewell™ 800, 24-well plate	STEMCELL Technologies	Cat# 34815
Anti-Adherence Rinsing Solution	STEMCELL Technologies	Cat# 07010
QIAzol lysis reagent	Qiagen	Cat# 79306
RNeasy® Mini Kit	Qiagen	Cat# 74106
iScript™ gDNA Clear cDNA Synthesis Kit	Bio-Rad	Cat# 1725035
SsoFast™ EvaGreen® Supermix	Bio-Rad	Cat# 1725202
UltraPure DNase/RNase-Free Distilled Water	Life technologies	Cat# 10977-015
Dithizone	Sigma-Aldrich	Cat# 43820-50G
4% Paraformaldehyde (PFA) solution in PBS	Santa Cruz Biotechnology	Cat# sc-281692
100% Triton X-100	Thermo Fisher Scientific	Cat# BP151-100
Bovine serum albumin (BSA)	Thermo Fisher Scientific	Cat# BP1600-100
Cell-Tak Cell and Tissue Adhesive	Corning	Cat# 354240
**Experimental cell lines**		
Human: H1 hESCs	WiCell Research Institute	James A. Thomson et al., 1998
Human: Mel1 INS$^{GFP/W}$ hESCs	Kindly provided by Dr. Edouard G. Stanley	Suzanne J Micallef et al., 2011
Human: HUES4 PDXeG hESCs	Kindly provided by Dr. Henrik Semb	Jacqueline Ameri et al., 2017
Human: GCaMP_CRISPRi hiPSCs	Kindly provided by Dr. Bruce Conklin and Dr. Knut Woltjen	Mohammad A. Mandegar et al., 2016
Human: HUES8 iCas9 hESCs	Kindly provided by Dr. Danwei Huangfu	Zengrong Zhu et al., 2016
Human: HUES8 PDX1-/- KO hESCs	Kindly provided by Dr. Danwei Huangfu	Zengrong Zhu et al., 2016
Human: HUES8 RFX6-/- KO hESCs	Kindly provided by Dr. Danwei Huangfu	Zengrong Zhu et al., 2016
**Oligonucleotides**		
See Table S3 for primer information	Integrated DNA Technologies	https://www.idtdna.com/pages/
**Software and web-based platforms**		
GraphPad Prism 10.0	GraphPad	https://www.graphpad.com/
Fiji/ImageJ	Schneider et al., 2012	https://imagej.nih.gov/ij/
FlowJo v10	FlowJo, LLC	http://www.flowjo.com
BioRender	BioRender	https://app.biorender.com
RStudio	RStudio Team	http://www.rstudio.com/
Heatmapper	Sasha Babicki et al., 2016	http://www.heatmapper.ca
NetworkAnalyst	Ewald J. et al., 2023	https://www.networkanalyst.ca
PlotTwist	Goedhart, J., 2020	https://huygens.science.uva.nl

Supplementary Table 3: The sequences of qPCR primers used in this study.

Target	Primer	Sequence (from 5' to 3')
PDX1	forward	TTTCCCATGGATGAAGTCTACCAAA
	reverse	GTGAGATGTACTTGTTGAATAGGAACTC
NKX6.1	forward	CCTGTACCCCTCATCAAGGAT
	reverse	CAAGTATTTTGTTTGTTCGAAAGTCTTCT
NEUROD1	forward	GGTTATGAGACTATCACTGCTCAG
	reverse	AGAACTGAGACACTCGTCTGTC
CHGA	forward	CCTGTGAACAGCCCTATGAATAAAG
	reverse	CTGATGTCTCAGAATGGAAAGGATC
ISL1	forward	AATGTGCGGAGTGTAATCAGTATTTG
	reverse	CATTTGATCCCGTACAACCTGATATAATC
INS	forward	GCAGCCTTTGTGAACCAACA
	reverse	GGTGTGTAGAAGAAGCCTCGTT
GCG	forward	TTCTACAGCACACTACCAGAAGA
	reverse	CTGGGAAGCTGAGAATGATCTG
SOX9	forward	GGAACAACCCGTCTACACACA
	reverse	CTTTCTTCGGTTATTTTTAGGATCATCTC
KRT18	forward	GAGACTGGAGCCATTACTTCAAG
	reverse	CTAAAGTCATCAGCAGCAAGACG
MUC1	forward	GATACCTACCATCCTATGAGCGAG
	reverse	CTGCTGGGTTTGTGTAAGAGAG
SPP1	forward	ATTCTGGAAGTTCTGAGGAAAAGC
	reverse	GTCATTGGTTTCTTCAGAGGACAC
VIM	forward	CAGATTACAGAATGGAGACAGAAAGC
	reverse	GTTCTGATGCTTAGACAGGACTTTG
THY1	forward	GTCACAGTGCTCAGAGACAAAC
	reverse	CCAGTCACAGGGACATGAAATC
COL1A1	forward	GTGATTGAATACAAAACCACCAAGAC
	reverse	GAGTTTACAGGAAGCAGACAGG
PBX1	forward	ATATTTCTATTCCCATCTCAGCAACC
	reverse	CGCTTATTTCCAAACCAGTTTGATAC
PBX3	forward	CACACAGAACTGGAGAAATATGAACA
	reverse	CACCATTCTTTCAATCTCTTTTGGAG

Target	Primer	Sequence (from 5' to 3')
PCNA	forward	TCACAGACAAGTAATGTCGATAAAGAG
	reverse	GAGTGGCTTTTGTAAAGAAGTTCAG
SLIT1	forward	GTGGTGCTTTTGATGACATGAAG
	reverse	CACTCAAGTCCAGTCTTGACAAAG
SLIT2	forward	CATCCAGCTTCTGCCATTTACA
	reverse	CTTTGACAAGTTAGTGATGATTTTGGG
ROBO2	forward	ACAGTCAAGGACAGTTTACAGGT
	reverse	CTGTTTAGAGTCATCACTTCCATGAG
CDH1	forward	CAGAATAAAGACCAAGTGACCACC
	reverse	GCAAGAGCAGCAGAATCAGAATTAG
EPHB1	forward	GAGAATGGTGCATTGGATTCTTTC
	reverse	CATAATTCATCTCAGCCAGGTACTTC
EPHB2	forward	GCTAGACAAGATGATCCGCAATC
	reverse	CACCGTGTTAAAGCTGGTGTAG
EPHB3	forward	GCTATCGTCTGCCTCAGGAAG
	reverse	CCTTCATTCCAGGAGCAATGTAC
EPHB4	forward	AGTATCTCATCGGACATGGTACTAAG
	reverse	CAATCACCTCTTCAATCTTGACGTA
EPHB6	forward	GAATAGCCACTTGGTGTGCAAG
	reverse	GATGTTGTATGCTTTCCATGTGC
EFNB1	forward	GCACCATGATTACTACATTACCTCAA
	reverse	CTCAGGCGTCACAGCATTG
EFNB2	forward	TCGACAACAAGTCCCTTTGTAAAAC
	reverse	CCTGAAGCAATCCCTGCAAATAAG
EFNB3	forward	CACCACGATTACTACATCATTGCC
	reverse	ACCTTCATGCCTCTGGTTAGG
NGN3	forward	ACCACCCCATAATCTCATTCAAAG
	reverse	GTAAGAGACTGAGAGGCAGACAG
SST	forward	TCCGTCAGTTTCTGCAGAAGTC
	reverse	CTGGGACAGA TCTTCAGGTTCC
PPY	forward	GCCTAGGTATGGGAAAAGACACA
	reverse	TCATGGAGTCGTAGGAGACAGAA

Target	Primer	Sequence (from 5' to 3')
GHRL	forward	GAAGATGGAGGTCAAGCAGAAG
	reverse	CTGTGCTGCTGGTACTGAAC
SLC18A1	forward	GTACCAGCTTGCTCTGTGTTC
	reverse	GGCATCATAGAAGAATCCACCAT
MAFA	forward	GTGAGTCCTGTGCTCAGTCG
	reverse	CTCTTGAAGGTTAAAACAAGATGTATTTCC
MAFB	forward	GACTCCTGGCTTTCTGAACTTTG
	reverse	CTCTCCTTTCCTCGTTGCTCTC
ARX	forward	CTCAGCACCACTCAAGACCAA
	reverse	GCATCCAGACTGCTGTGAAG
HHEX	forward	CAGCGAGAGACAGGTCAAAAC
	reverse	ATCACAGGAACTGTCCAAACTTTC
NKX2.2	forward	CACGAA TTGACCAAGTGAAGC
	reverse	GGCAGGTCTAAGATGTCCTTG
IAPP	forward	ATCTGAAAGCTACACCCATTGAAAG
	reverse	AGAGAATGGCACCAAAGTTGTTG
ABCC8	forward	GACACCAATCAGCTCATGTGG
	reverse	GCACTGACTCCGAGTATGTAGTAG
GCK	forward	TGAGGCACGAAGACATCGATAAG
	reverse	TTGCCACCACATCCATTTCAAAG
KCNK1	forward	GAAGGCTACAATCAAAAATTCAGAGAG
	reverse	CTCATGGAGTTCACAGAAGGTTTC
KCNK3	forward	CAGAAGACTTCAGACTCACCATAATTG
	reverse	CTAAGTAGGGTTTTGTGAGACTCAAAG
PCSK1	forward	CTCTGAAGACAGTCTGTATAATGACTATG
	reverse	GGAAGAAGCATGAATATTTCCAACTTG
PCSK2	forward	CAGGCAATTCCGTCTTCTTAATCTG
	reverse	GAATTTAGAGTCACACATAGGACATGG
NFX1	forward	TTTCAGAACAAAGGAGCTTCCAT
	reverse	CTTATCCACACAGCATATCTCATTACA
