# Supplementary material for: PDX1+ cell budding morphogenesis in a stem cell-derived islet spheroid system
Source: Nat Commun. 2024 Jul 13;15:5894. doi: 10.1038/s41467-024-50109-2 (PMC11246529; doi:10.1038/s41467-024-50109-2)
Supplement: Supplementary file 3 — Description of Additional Supplementary Files [file 41467_2024_50109_MOESM3_ESM.pdf]

Description of Additional Supplementary Files

File Name: Supplementary Movie 1
Description: **In-chip calcium imaging of representative Stage 6 islet bud.** The Stage 6 islet bud was stained with Cal-520 AM dye (a calcium indicator), loaded into microfluidic chip and staged on spinning-disc confocal microscope for live-cell calcium imaging. During imaging, islet was kept at 37°C and sequentially perfused with low glucose KRB buffer (containing 3.3 mM glucose) for 5 min and high glucose KRB buffer (containing 16.7 mM glucose) for 10 min. Laser with 488 nm wavelength was used to illuminate Cal-520 signals and emission was collected using a single band pass filter (525 nm wavelength, 50 nm band width). Timelapse images were captured very 0.6 s with 100 ms exposure time under 40x/1.25 NA objective using SlideBook 6 software.

File Name: Supplementary Movie 2
Description: **In-chip calcium imaging of representative Stage 7 islet bud.** The Stage 7 islet bud was stained with cell permeable calcium dye Cal-520 AM, loaded into microfluidic chip and staged on the spinning-disc confocal microscope. During imaging, islet was kept at 37°C and sequentially perfused with low glucose KRB buffer (containing 3.3 mM glucose) for 5 min and high glucose KRB buffer (containing 16.7 mM glucose) for 20 min. Laser with 488 nm wavelength was used to illuminate Cal-520 signals and emission was collected using a single band pass filter (525 nm wavelength, 50 nm band width). Timelapse images were captured very 0.6 s with 100 ms exposure time under 40x/1.25 NA objective using SlideBook 6 software.
