# Supplementary material for: PDX1+ cell budding morphogenesis in a stem cell-derived islet spheroid system
Source: Nat Commun. 2024 Jul 13;15:5894. doi: 10.1038/s41467-024-50109-2 (PMC11246529; doi:10.1038/s41467-024-50109-2)
Supplement: Supplementary file 6 — Reporting Summary [file 41467_2024_50109_MOESM6_ESM.pdf]

nature portfolio

Corresponding author(s): Timothy Kieffer; Jia Zhao

Last updated by author(s): 2024-5-10

Reporting Summary

Nature Portfolio wishes to improve the reproducibility of the work that we publish. This form provides structure for consistency and transparency in reporting. For further information on Nature Portfolio policies, see our Editorial Policies and the Editorial Policy Checklist.

Statistics

For all statistical analyses, confirm that the following items are present in the figure legend, table legend, main text, or Methods section.

n/a	Confirmed	
☐	☒	The exact sample size (*n*) for each experimental group/condition, given as a discrete number and unit of measurement
☐	☒	A statement on whether measurements were taken from distinct samples or whether the same sample was measured repeatedly
☐	☒	The statistical test(s) used AND whether they are one- or two-sided<br>*Only common tests should be described solely by name; describe more complex techniques in the Methods section.*
☒	☐	A description of all covariates tested
☐	☒	A description of any assumptions or corrections, such as tests of normality and adjustment for multiple comparisons
☐	☒	A full description of the statistical parameters including central tendency (e.g. means) or other basic estimates (e.g. regression coefficient) AND variation (e.g. standard deviation) or associated estimates of uncertainty (e.g. confidence intervals)
☐	☒	For null hypothesis testing, the test statistic (e.g. *F*, *t*, *r*) with confidence intervals, effect sizes, degrees of freedom and *P* value noted<br>*Give P values as exact values whenever suitable.*
☒	☐	For Bayesian analysis, information on the choice of priors and Markov chain Monte Carlo settings
☒	☐	For hierarchical and complex designs, identification of the appropriate level for tests and full reporting of outcomes
☒	☐	Estimates of effect sizes (e.g. Cohen's *d*, Pearson's *r*), indicating how they were calculated

Our web collection on statistics for biologists contains articles on many of the points above.

Software and code

Policy information about availability of computer code

Data collection	Detailed description on data collection can be found in the Methods section.
Data analysis	GraphPad Prism version 10 software for statistical analysis; FlowJo version 10.1 software for flow cytometry analysis; Leica LAS, ImageXpressMicro, Zeiss Axio Zoom version 16 and SlideBook 6 softwares for capturing images; Fiji and MetaXpress softwares for image processing and analysis; STAR (Aligner) 2.5.0b version, Cufflinks version 2.2.1 softwares for bulk RNA-seq data alignment and assembly; DESeq2, ComplexHeatmap and clusterProfiler R packages for RNA-seq data analysis; BioRender, Heatmapper, Network Analyst and PlotTwist web applications for generating graphs.

For manuscripts utilizing custom algorithms or software that are central to the research but not yet described in published literature, software must be made available to editors and reviewers. We strongly encourage code deposition in a community repository (e.g. GitHub). See the Nature Portfolio guidelines for submitting code & software for further information.

Data

Policy information about availability of data

All manuscripts must include a data availability statement. This statement should provide the following information, where applicable:

- Accession codes, unique identifiers, or web links for publicly available datasets
- A description of any restrictions on data availability
- For clinical datasets or third party data, please ensure that the statement adheres to our policy

All data that support the findings of this study are available in a publicly accessible repository and provided in Source Data file as well as in the Data/Code Availability statements in the manuscript. Specifically, RNA sequencing data generated in this study was deposited to the NCBI Gene Expression Omnibus (GEO) under accession number GSE249020. Source data are provided with this paper. Refer to the Source Data file for all data reported in this study. Code for data analysis was deposited to the GitHub (https://github.com/hcen/RNAseq_budding), which includes all information required to reanalyze the data.

Research involving human participants, their data, or biological material

Policy information about studies with human participants or human data. See also policy information about sex, gender (identity/presentation), and sexual orientation and race, ethnicity and racism.

Reporting on sex and gender	The H1 hESC line (male) was obtained from WiCell; Mel1 INSGFP/W line (male) was provided by Dr. Edouard G. Stanley from MCRI and Monash University; HUES4 PDXeG line (male) was provided by Dr. Henrik Semb; GCaMP_CRISPRi hiPSC line (male) was provided by Dr. Knut Woltjen and Dr. Bruce Conklin. HUES8 iCas9 parental, PDX1 KO and RFX6 KO lines (male) were provided by Dr. Danwei Huangfu.
Reporting on race, ethnicity, or other socially relevant groupings	See above.
Population characteristics	See above.
Recruitment	As we did not generate these cells, we were not part of the recruitment to collect donor materials.
Ethics oversight	All work involving human pluripotent stem cells and human islets carried out in this study were approved by the University of British Columbia Clinical Research Ethics Board and the Canadian Stem Cell Oversight Committee with appropriate consent and conditions.

Note that full information on the approval of the study protocol must also be provided in the manuscript.

Field-specific reporting

Please select the one below that is the best fit for your research. If you are not sure, read the appropriate sections before making your selection.

☒ Life sciences ☐ Behavioural & social sciences ☐ Ecological, evolutionary & environmental sciences

For a reference copy of the document with all sections, see nature.com/documents/nr-reporting-summary-flat.pdf

Life sciences study design

All studies must disclose on these points even when the disclosure is negative.

Sample size	For all experiments, at least three independent biological replicates were performed to confirm phenotypes, use for statistical quantification and ensure the reproducibility of our findings.
Data exclusions	For bulk RNA-seq data, genes detected with < 5 raw counts in < 25% samples were filtered out. Beyond this, no data were excluded.
Replication	We performed multiple independent experiments and all experiments were successfully repeated a minimum of three times with biological replicates to ensure the reproducibility of our findings.
Randomization	The allocation of samples into experimental groups were based on genotypes (e.g., wildtype or specific gene knockout) or compound treatment conditions (e.g., vehicle control or test group). All samples were selected at random from differentiation batches.
Blinding	For qPCR experiments, samples were blinded to technical personnels. For other experiments, investigators were not blinded during data acquisition.

Reporting for specific materials, systems and methods

We require information from authors about some types of materials, experimental systems and methods used in many studies. Here, indicate whether each material, system or method listed is relevant to your study. If you are not sure if a list item applies to your research, read the appropriate section before selecting a response.

Materials & experimental systems

n/a	Involved in the study
☐	☒ Antibodies
☐	☒ Eukaryotic cell lines
☒	☐ Palaeontology and archaeology
☒	☐ Animals and other organisms
☒	☐ Clinical data
☒	☐ Dual use research of concern
☒	☐ Plants

Methods

n/a	Involved in the study
☒	☐ ChIP-seq
☐	☒ Flow cytometry
☒	☐ MRI-based neuroimaging

Antibodies

Antibodies used	See Table S2 and the Methods section for details on antibody information (including vendor and catalogue numbers) and dilutions.
Validation	All the commercial antibodies used in this study are validated by the vendors and in multiple research publications.<br>Specifically, antibodies from Cell Signaling Technologies are routinely tested and validated by the manufacturer (https://www.cellsignal.com/about-us/our-approach-process/cst-antibody-performance-guarantee).<br>Antibodies from R&D Systems and Novus Biologicals are validated in-house to ensure the validity and specificity (https://www.rndsystems.com/quality/antibodies-built-for-reproducibility).<br>Antibodies from Abcam applies an in-house validation strategy incorporating routine use of knockout studies to provide negative controls and KO validation (https://www.abcam.com/help/abpromise-guarantee).<br>Antibodies from Millipore Sigma are validated with enhanced validation strategies to ensure reproducibility, specificity and performance (https://www.sigmaaldrich.com/CA/en/technical-documents/technical-article/protein-biology/immunohistochemistry/antibody-enhanced-validation).<br>Antibodies from DSHB are validated by both the manufacturer and the Beta Cell Consortium (http://www.betacell.org/ab/ab_view).<br>Antibodies from BD Biosiences are routinely QC tested and validated by the manufacturer to ensure specificity and reproducibility (https://www.bdbiosciences.com/en-eu/products/reagents/flow-cytometry-reagents/research-reagents/quality-and-reproducibility).

Eukaryotic cell lines

Policy information about cell lines and Sex and Gender in Research

Cell line source(s)	The H1 hESC line (male) was obtained from WiCell; Mel1 INSGFP/W line (male) was provided by Dr. Edouard G. Stanley from MCRI and Monash University; HUES4 PDXeG line (male) was provided by Dr. Henrik Semb; GCaMP_CRISPRi hiPSC line (male) was provided by Dr. Knut Woltjen and Dr. Bruce Conklin. HUES8 iCas9 parental, PDX1 KO and RFX6 KO lines (male) were provided by Dr. Danwei Huangfu.
Authentication	We performed directed differentiation from these cell lines above into pancreatic progenitors and insulin-producing cell lines. The reliability of insulin and PDX1 reporter lines were validated in original research publications as well as in our own hands as shown in Figure S19. All the other cell lines were rigorously validated by the original research publications.
Mycoplasma contamination	We performed routine mycoplasma tests using a PCR-based method and all cell lines used in this study were found to be negative for mycoplasma.
Commonly misidentified lines<br>(See ICLAC register)	None.

Plants

Seed stocks	N/A.
Novel plant genotypes	N/A.
Authentication	N/A.

Flow Cytometry

Plots

Confirm that:

☒ The axis labels state the marker and fluorochrome used (e.g. CD4-FITC).

☒ The axis scales are clearly visible. Include numbers along axes only for bottom left plot of group (a 'group' is an analysis of identical markers).

☒ All plots are contour plots with outliers or pseudocolor plots.

☒ A numerical value for number of cells or percentage (with statistics) is provided.

Methodology

Sample preparation	Stages 1 and 4-7 cultures were dissociated, fixed, permeabilized and stained for various intracellular markers. More details can be found in the Methods section.
Instrument	CytoFLEX flow cytometer (Beckman Coulter)
Software	CytExpert software and FlowJo v. 10.1 software (FlowJo, LLC)
Cell population abundance	GFP+ cells were enriched to 70-90% post flow sorting.
Gating strategy	All captured events were first plotted by forward scatter area (FSC-A) and side scatter area (SSC-A). In this plot, debris were removed and cells were identified. Next, singlets were identified by gating based on the plots by forward scatter area (FSC-A) and forward scatter width (FSC-W). Finally, flow cytometry plots were gated by unstained and/or isotype controls on fluorophore channels. An example showing the gating strategy is provided in Figure S20.

☒ Tick this box to confirm that a figure exemplifying the gating strategy is provided in the Supplementary Information.
